# Supplementary figures and images for: Suicide attempt and death by suicide among parents of young individuals with cancer: A population-based study in Denmark and Sweden
Source: PLoS Med. 2024 Jan 16;21(1):e1004322. doi: 10.1371/journal.pmed.1004322 (PMC10791002; doi:10.1371/journal.pmed.1004322)

**Figure S1. Flow chart of the study design**

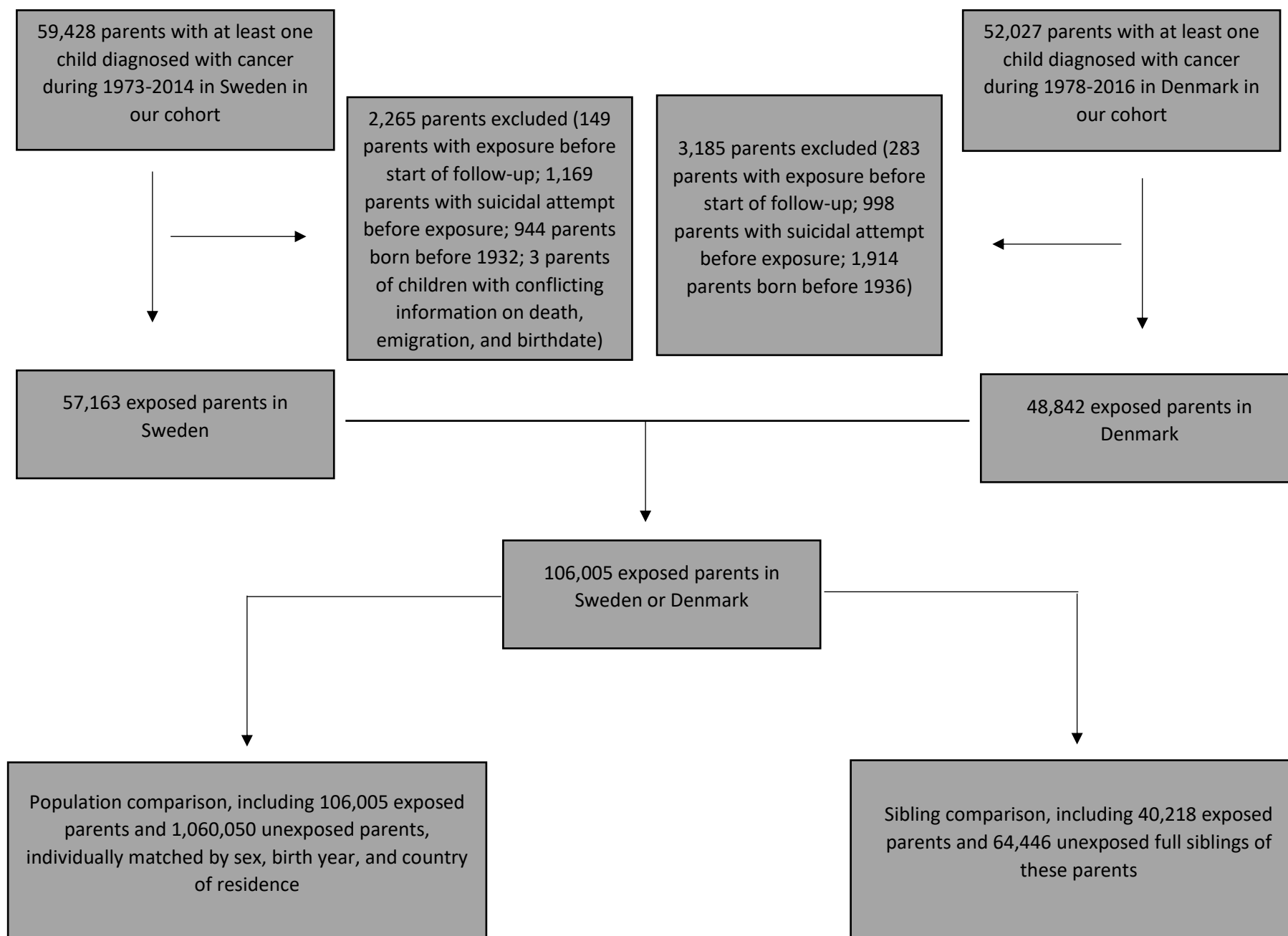

Supplement: S1 Fig — (PDF) [file pmed.1004322.s001.pdf]
