# Supplementary material for: Suicide attempt and death by suicide among parents of young individuals with cancer: A population-based study in Denmark and Sweden
Source: PLoS Med. 2024 Jan 16;21(1):e1004322. doi: 10.1371/journal.pmed.1004322 (PMC10791002; doi:10.1371/journal.pmed.1004322)

S2 Fig. Unadjusted hazard ratio (HR) with 95% confidence interval (CI) of suicide attempt and death by suicide in relation to cancer diagnosis of a child, analyses of population or sibling comparison

HR and associated 95% CI were estimated from flexible parametric survival models, allowing the effect of cancer diagnosis of a child to vary over time. A spline with 5 degrees of freedom (4 intermediate knots and 2 knots at each boundary, placed according to quintile distribution of events) was used for the baseline rate, while a spline with 3 degrees of freedom was used for the time-varying effect. (A), (B), (C) and (D) were without any adjustment. The analyses of flexible parametric survival models were performed on five imputed datasets and HRs were obtained from combination of each dataset using Rubin's rule.

A. Suicide attempt in population comparison

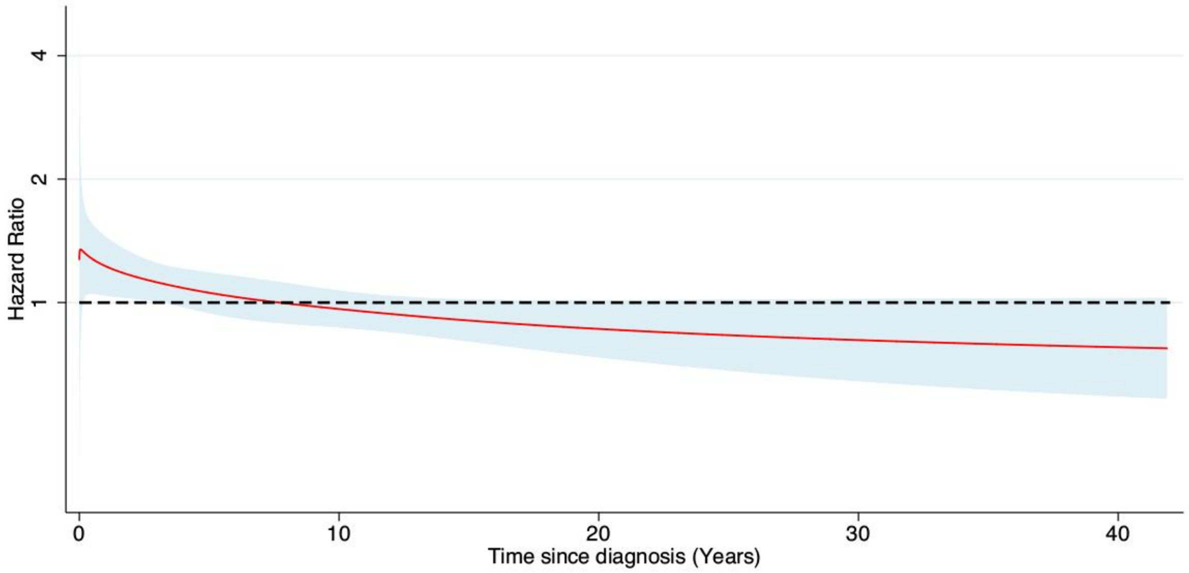

B. Suicide attempt in sibling comparison

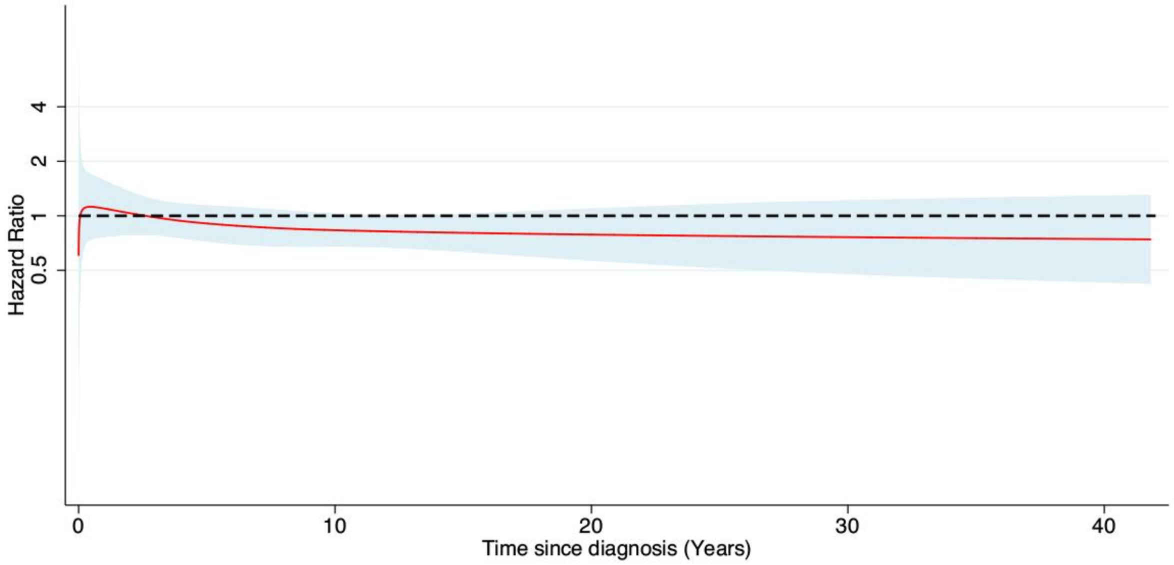

C. Death by suicide in population comparison

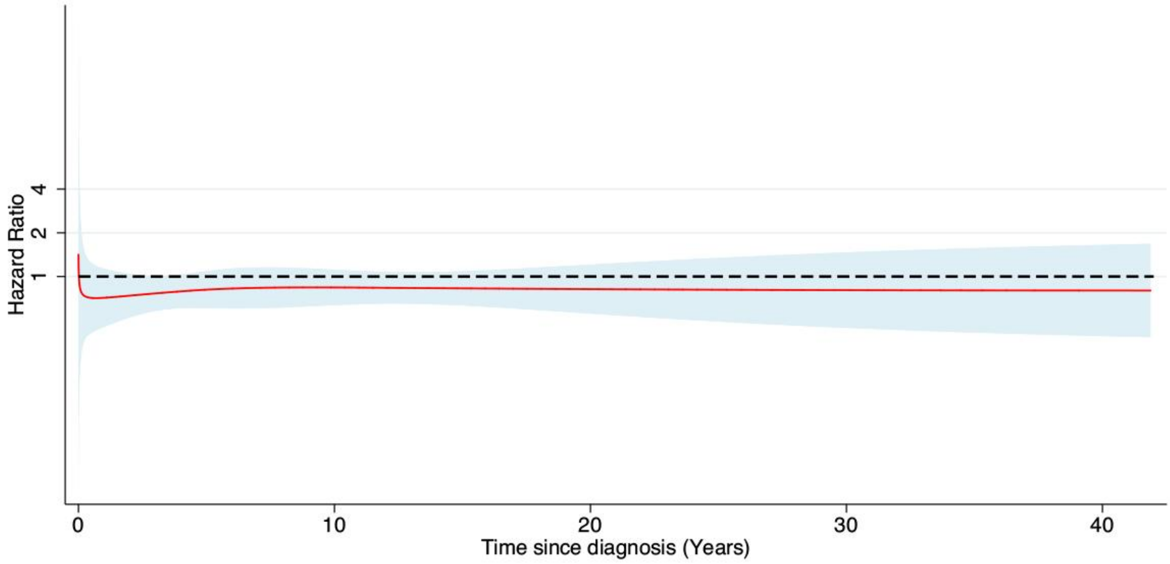

D. Death by suicide in sibling comparison

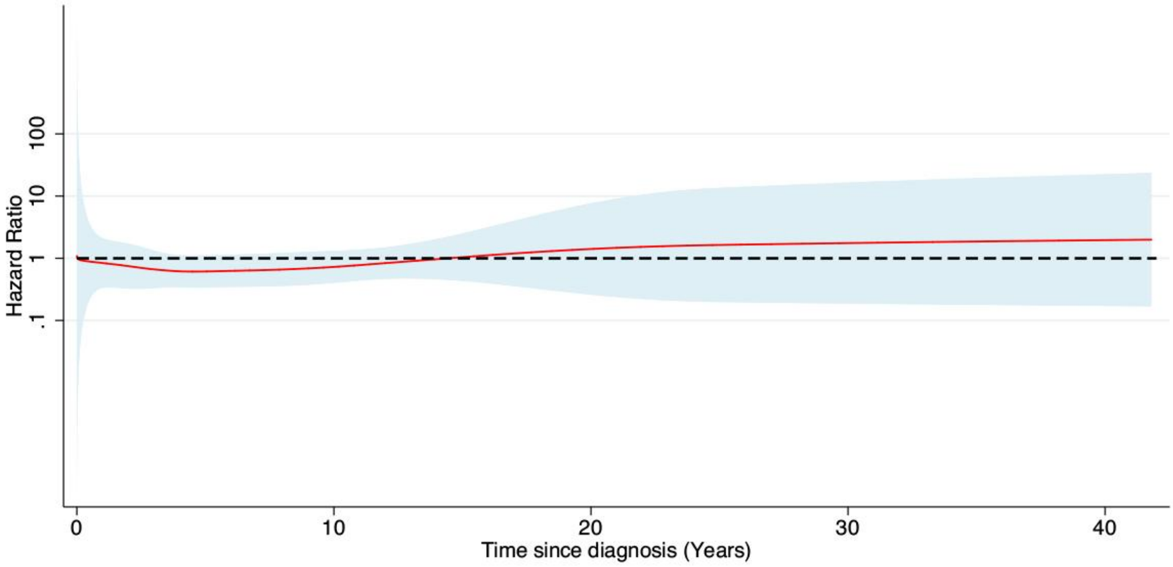

Supplement: S2 Fig — HR and associated 95% CI were estimated from flexible parametric survival models, allowing the effect of cancer diagnosis of a child to vary over time. A spline with 5 degrees of freedom (4 intermediate knots and 2 knots at each boundary, placed according to quintile distribution of events) was used for the baseline rate, while a spline with 3 degrees of freedom was used for the time-varying effect. (A), (B), (C), and (D) were without any adjustment. The analyses of flexible parametric survival models were performed on 5 imputed datasets, and HRs were obtained from combination of each dataset using Rubin’s rule. (A) Suicide attempt in population comparison. (B) Suicide attempt in sibling comparison. (C) Death by suicide in population comparison. (D) Death by suicide in sibling comparison. (PDF) [file pmed.1004322.s002.pdf]
