# Supplementary material for: Suicide attempt and death by suicide among parents of young individuals with cancer: A population-based study in Denmark and Sweden
Source: PLoS Med. 2024 Jan 16;21(1):e1004322. doi: 10.1371/journal.pmed.1004322 (PMC10791002; doi:10.1371/journal.pmed.1004322)

S3 Fig. Hazard ratio (HR) with 95% confidence interval (CI) of suicide attempt and suicide by death in relation to cancer diagnosis of a child, analyses among parents with a history of suicide attempt

HR and associated 95% CI were estimated from flexible parametric survival models, allowing the effect of cancer diagnosis of a child to vary over time. A spline with 5 degrees of freedom (4 intermediate knots and 2 knots at each boundary, placed according to quintile distribution of events) was used for the baseline rate, while a spline with 3 degrees of freedom was used for the time-varying effect. (A) and (C) were without any adjustment. (B) and (D) were adjusted for sex, age at cohort entry, country of residence, calendar year at cohort entry, marital status, the highest attained education, personal income, history of cancer, history of psychiatric disorder, and family history of psychiatric disorder. The analyses of flexible parametric survival models were performed on five imputed datasets and HRs were obtained from combination of each dataset using Rubin's rule.

A.Suicide attempt in unadjusted model

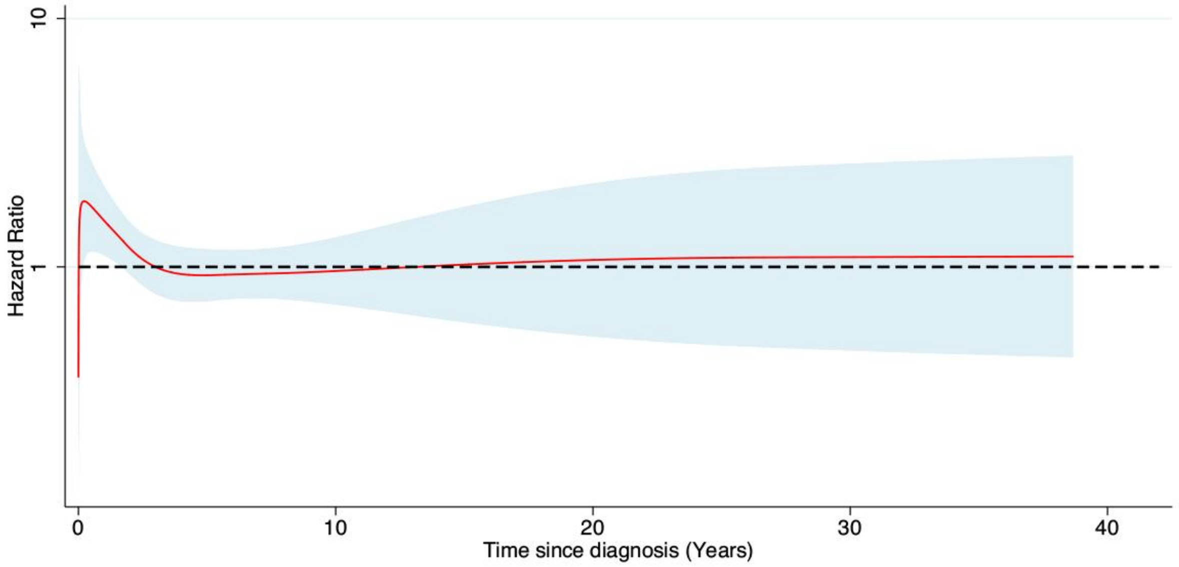

B. Suicide attempt in adjusted model

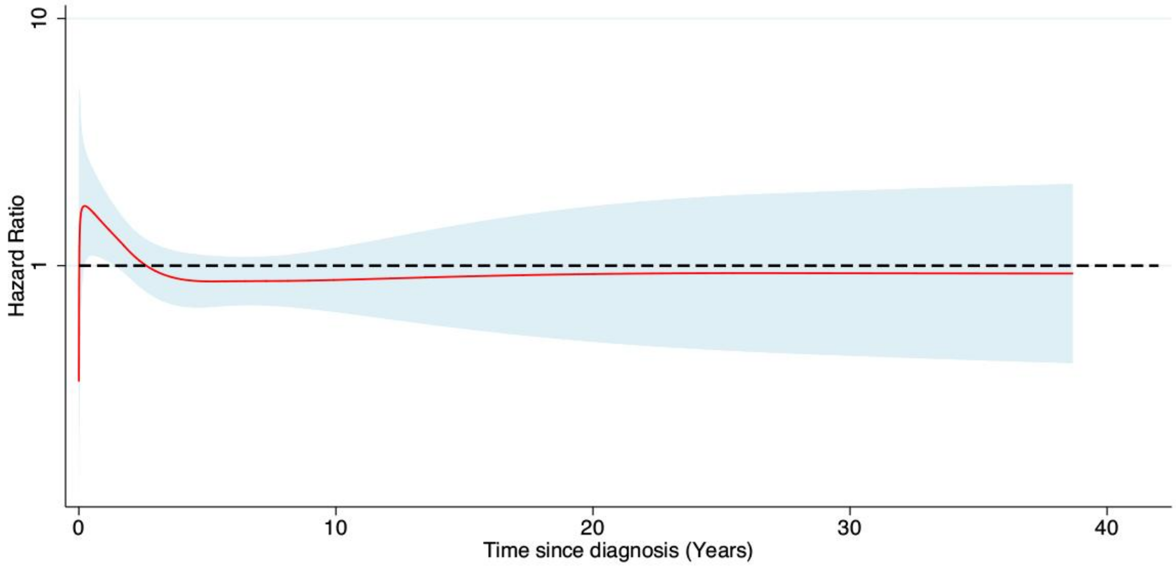

C. Death by suicide in unadjusted model

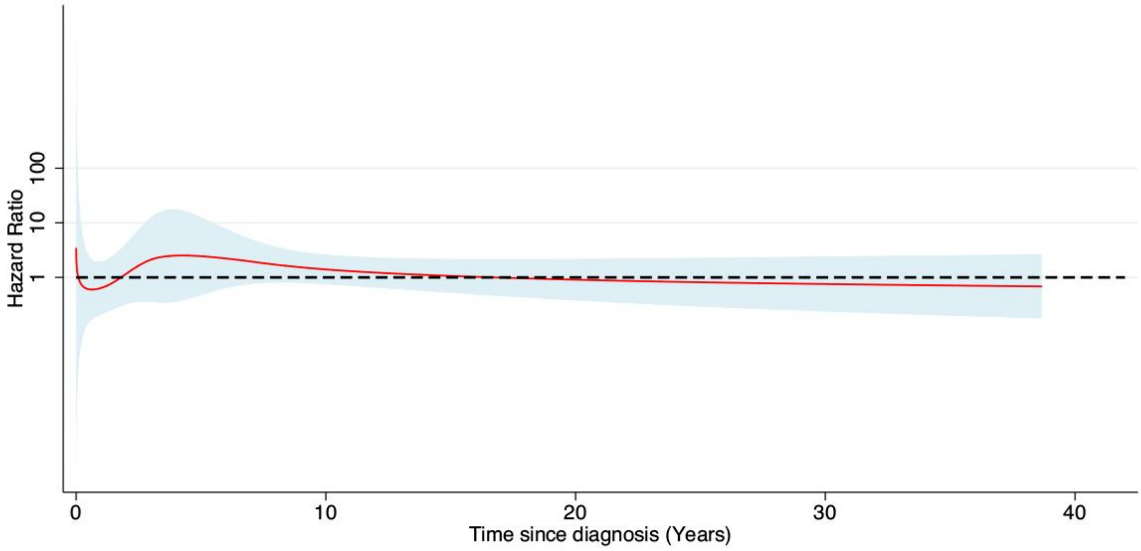

D. Death by suicide in adjusted model

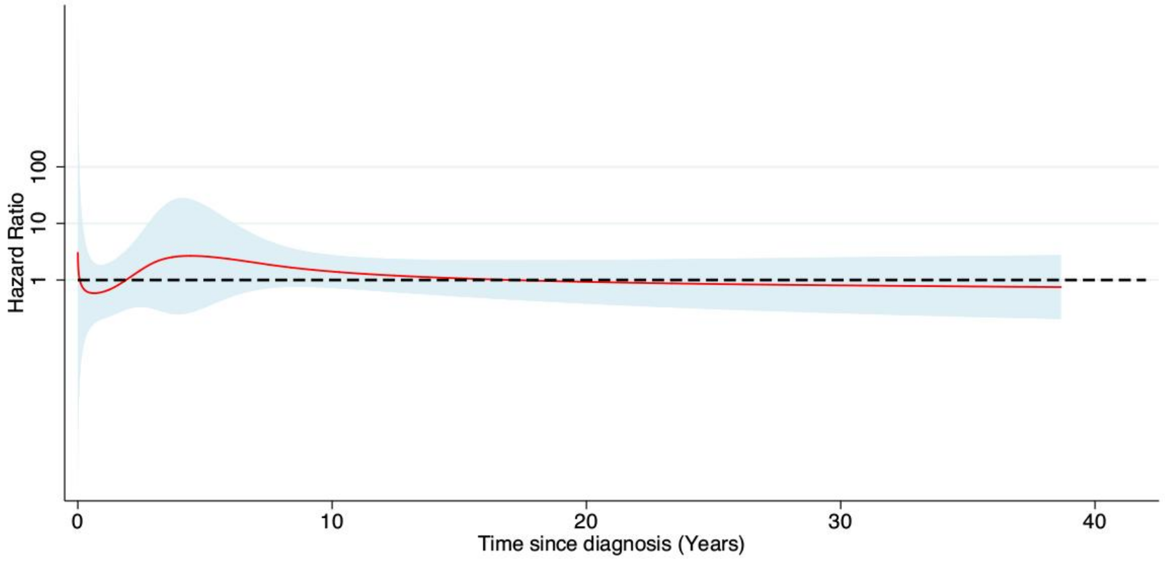

Supplement: S3 Fig — HR and associated 95% CI were estimated from flexible parametric survival models, allowing the effect of cancer diagnosis of a child to vary over time. A spline with 5 degrees of freedom (4 intermediate knots and 2 knots at each boundary, placed according to quintile distribution of events) was used for the baseline rate, while a spline with 3 degrees of freedom was used for the time-varying effect. (A) and (C) were without any adjustment. (B) and (D) were adjusted for sex, age at cohort entry, country of residence, calendar year at cohort entry, marital status, the highest attained education, personal income, history of cancer, history of psychiatric disorder, and family history of psychiatric disorder. The analyses of flexible parametric survival models were performed on 5 imputed datasets, and HRs were obtained from combination of each dataset using Rubin’s rule. (A) Suicide attempt in unadjusted model. (B) Suicide attempt in adjusted model. (C) Death by suicide in unadjusted model. (D) Death by suicide in adjusted model. (PDF) [file pmed.1004322.s003.pdf]
