# Supplementary material for: Suicide attempt and death by suicide among parents of young individuals with cancer: A population-based study in Denmark and Sweden
Source: PLoS Med. 2024 Jan 16;21(1):e1004322. doi: 10.1371/journal.pmed.1004322 (PMC10791002; doi:10.1371/journal.pmed.1004322)
Supplement: S1 Table — (PDF) [file pmed.1004322.s004.pdf]

S1 Table. Danish and Swedish revisions of the International Classification of Diseases (ICD) codes for cancer subtypes in the Danish and Swedish Cancer Registers[a]

Cancer characteristics	ICD-7	ICD-10
**Cancer type**		
Overall	140-207	C00-C97
Central nervous system	193	C70-C72
Hematological malignancy	200-207	C81-C96
Other cancer types	Other codes except for codes of cancer in central nervous system and hematological malignancy	Other codes except for codes of cancer in central nervous system and hematological malignancy
**Cancer aggressiveness[b]**		
Low	170, 172, 177, 190, 191, 194	C43, C44, C50, C54, C61, C73
Medium	Other codes except for codes of low aggressiveness and high aggressiveness	Other codes except for codes of low aggressiveness and high aggressiveness
High	150, 156, 157, 162, 163, 199	C15, C22-C25, C33-C34, C56, C76, C80

[a] We used both ICD-7 and ICD-10 in the Danish Cancer Register and only ICD-7 in Swedish Cancer Register to identify cancer cases.

[b] Cancer aggressiveness was defined according to 5-year survival rate of each cancer type (https://www-dep.iarc.fr/nordcan/english/frame.asp.). Low aggressiveness of cancer type includes breast cancer, prostate cancer, non-melanoma skin cancer, melanoma, corpus uteri cancer and thyroid cancer. High aggressiveness of cancer type includes lung cancer, oesophagus cancer, liver cancer, pancreatic cancer, ovary cancer and unknown/illed-defined cancer. Medium aggressiveness of cancer type includes other cancer types not included in low aggressiveness and high aggressiveness.
