# Supplementary material for: Suicide attempt and death by suicide among parents of young individuals with cancer: A population-based study in Denmark and Sweden
Source: PLoS Med. 2024 Jan 16;21(1):e1004322. doi: 10.1371/journal.pmed.1004322 (PMC10791002; doi:10.1371/journal.pmed.1004322)
Supplement: S2 Table — (PDF) [file pmed.1004322.s005.pdf]

S2 Table. Danish and Swedish revisions of the International Classification of Diseases (ICD) codes for suicide attempt and psychiatric disorder in the Patient Register and the Psychiatric Central Register in Denmark and Patient Register in Sweden, and death by suicide in the Danish Causes of Death Register and the Swedish Causes of Death Register

Suicidal behaviors	Denmark	Sweden
**Suicidal attempt**	ICD-8[a]: E950-E959<br>ICD-10: X60-X84, T39, T42, T43, T58	ICD-8: E950-E959<br>ICD-9: E950-E959<br>ICD-10: X60-X84, Y870
**Death by suicide**	ICD-8: 950-959<br>ICD-10: X60-X84	ICD-8: 950-959<br>ICD-9: 950-959<br>ICD-10: X60-X84, Y870
**Psychiatric disorder**	ICD-8: 291, 295-315<br>ICD-10: F10-F99	ICD-8: 291, 295-315<br>ICD-9: 291, 292, 295-319<br>ICD-10: F10-F99

[a]In addition to ICD codes, the reason for contact code '4' (deliberate self-harm) was also used to identify suicide attempt in Denmark.
