# Supplementary material for: Suicide attempt and death by suicide among parents of young individuals with cancer: A population-based study in Denmark and Sweden
Source: PLoS Med. 2024 Jan 16;21(1):e1004322. doi: 10.1371/journal.pmed.1004322 (PMC10791002; doi:10.1371/journal.pmed.1004322)
Supplement: S1 Text — (PDF) [file pmed.1004322.s007.pdf]

**Analysis Plan--suicidal behavior among parents of children with cancer**

Qianwei Liu

Created at 2021.11.01

**Objective**

The overall aim of the study is to investigate the association between cancer diagnosis of children and suicidal behaviors of their parents.

The primary objective of the study is to investigate risk of suicidal attempt and completed suicide among parents of children with cancer and to investigate

1) The temporal pattern of the risk;
2) Whether the risk differ by parental or child's characteristics;

**Study Population**

In this binational study, we will first identify biological parents of all live births during 1973-2016 according to the Danish Medical Birth Register (MBR) and during 1973-2014 according to the Swedish MBR. As the parents might have had other children born before the establishment of the MBRs or born outside Denmark or Sweden, we will identify additional children of these parents through the Danish Civil Registration System and the Swedish Multi-Generation Register as well. The study period will be defined as 1978-2016 for Denmark and 1973-2014 for Sweden.

We will identify all parents who had a child diagnosed with cancer during the study period in Denmark or in Sweden. If a parent had more than one child with a cancer diagnosis, the date of cancer diagnosis of the first child will be regarded as the index exposure. As we aims to study newly diagnosed child cancer and first-onset parental suicidal attempt or completed suicide, parents that had a cancer child or suicidal attempt before the study period will be excluded from the main analysis. To ensure relatively complete ascertainment of familial links from the Danish Civil Registration System and Swedish Multi-Generation Register, we will exclude parents born before 1936 in Denmark or before 1932 in Sweden. The date of child cancer diagnosis will be defined as the index date for these exposed parents.

We will define two unexposed comparison groups. For each exposed parent, we will randomly select 10 parents from the study population, who were individually matched to the exposed parent on birth year, sex, and country of residence and had no history of suicidal attempt, at least one alive child, and no child with cancer, before the index date of the exposed parent, as population comparison. As cancer and suicidal behaviors might share risk factors including factors shared between family members, we will also perform a sibling comparison including the exposed parents and their unexposed full siblings who had no history of suicidal attempt and at least one child alive with no diagnosed cancer, before the index date of their exposed full sibling. We will use the index date of the exposed parent as the index date for their matched unexposed parents and unexposed siblings.

We will follow all study participants from the index date, until death, emigration, an incidence of suicidal attempt or completed suicide, or the end of follow-up (December 31st, 2016 in Denmark and December 31st, 2014 in Sweden), whichever came first. Follow-up of the unexposed parents and the unexposed siblings will be additionally censored if they had a child diagnosed with cancer during follow-up.

**Exposure**

We will use the Danish and Swedish revisions of the ICD codes to identify child cancer diagnoses from the Danish and Swedish Cancer Registers. We analyzed any cancer as well as common cancer types in childhood and early adulthood, including cancer in the CNS system, hematological malignancy, as well as other types. We will also perform the analysis by cancer aggressiveness.

**Outcome**

Suicidal attempt will be ascertained through the Patient Register and the Psychiatric Central Register in Denmark and the Patient Register in Sweden. Completed suicide will be identified through the Danish Civil Registration System and the Swedish Causes of Death Register, using the 8th and 10th Danish revisions and 8th, 9th, and 10th Swedish revisions of the ICD codes. The reason for contact code '4' will additionally be used to identify suicidal attempt in Denmark.

**Covariates**

We will collect information on sex, year of birth and marital status from the Danish Civil Registration System and the Swedish Total Population Register. We will collect information on household income from the Danish Integrated Database for Longitudinal Labor Market Research and the Swedish Register of Incomes and Taxes. We will collect information on the highest attained education from the Danish Integrated Database for Longitudinal Labour Market Research and the Swedish Education Register. We will collect information on history of cancer from the Cancer Registers in Denmark and Sweden, and on history of psychiatric disorder and family history of psychiatric disorder from the Danish Patient Register, the Danish Psychiatric Central Register, and the Swedish Patient Register. We will collect information on smoking (available since 1991 in Denmark and since 1982 in Sweden) and body mass index (BMI) (available since 2003 in Denmark and during 1982-1989 and since 1992 in Sweden) during early pregnancy from the MBRs (only available for mothers).

**Statistical Analysis**

We will first visualize the time-varying associations of child cancer diagnosis with the risk of parental suicidal attempt and completed suicide using flexible parametric survival models, in both the population and sibling comparisons. In the population comparison, we will adjust for sex, age at the index date, country of residence, calendar year of the index date, marital status, highest attained education, household income, history of cancer, history of psychiatric disorder, and family history of psychiatric disorder. In the sibling comparison, all models will be stratified by family identifier (mother's and father's identification numbers) and adjust for the same variables except family history of psychiatric disorder.

We will then analyze the effect of parental and child cancer characteristics on the association of interest. In these analyses, we will use Cox regression to estimate the hazard ratio (HR) and 95% confidence interval (CI) of suicidal attempt and completed suicide in relation to a child cancer diagnosis, with time since the index date as the underlying time scale and the same adjustment as described in flexible parametric survival models. To assess the effect of parental characteristics, we performed stratified analyses by sex, age at the index date (<40, 40 to 60, or >60), calendar

year of the index date (<1990, 1990 to 1999, 2000 to 2009, or ≥2010), country of residence, household income, marital status, highest attained education, history of cancer, history of psychiatric disorder, and family history of psychiatric disorder, as well as number of children at the time of child cancer diagnosis. We will assess the differences between the stratum-specific HRs by including interaction terms in Cox models and used Wald test to test the statistical significance of the interaction terms. To assess the effect of child cancer characteristics, we will perform subgroup analyses by child age at cancer diagnosis, cancer type (CNS cancer, hematological malignancy, or other types), cancer aggressiveness (low, medium, or high), as well as survival status of the cancer child.

**Sensitivity Analyses**

We will perform several sensitivity analyses to assess the robustness of the findings. To evaluate the effect of lifestyle factors on the association of interest, we will perform additional analyses after adjustment for smoking and BMI during pregnancy. We will also conduct a separate analysis, in which we will construct an additional cohort of parents (both exposed and unexposed) with at least one suicidal attempt before the index date, and use flexible parametric models to investigate the association of child cancer diagnosis with risk of recurrent suicidal attempt.

**Software**

The SAS 9.4 (SAS Institute) and Stata 15.1 (StataCorp LP) will be the primary analysis tool.

**Statistical Considerations**

If the association of interest remains stable with time in the flexible parametric model and the proportional hazard assumption holds across the whole follow-up, we will perform Cox Regression for the entire study period; if the association varies greatly with time and the proportional hazard assumption does not hold for the whole study period, we will performed Cox Regression by splitting the time period into different time periods by the cut-off of the values of hazard ratios. As some variables may have missing value, multiple imputation will be performed for missing values. Sensitivity analyses of complete case analysis and missing-indicator analysis will also be performed to test the robustness of multiple imputation.
