# Supplementary material for: Suicide attempt and death by suicide among parents of young individuals with cancer: A population-based study in Denmark and Sweden
Source: PLoS Med. 2024 Jan 16;21(1):e1004322. doi: 10.1371/journal.pmed.1004322 (PMC10791002; doi:10.1371/journal.pmed.1004322)
Supplement: S2 Text — (PDF) [file pmed.1004322.s008.pdf]

Supplementary methods of multiple imputation

To maximize statistical power and avoid selection bias, multiple imputation was performed to impute missing values. Fully conditional specification (FCS) method was applied to impute 5 complete datasets with 10 iterations for each dataset. In the main analysis, the proportion of covariates with missing data was: 1.7% for household income (below the lowest tertile, between the lowest and highest tertile, above the highest tertile), 1.5% for the highest attained education (≤9 years, 10-14 years, ≥15 years) and 18.5% for marital status (single, widowed or divorced, or married), respectively. We included all variables of the main analysis in the imputation model and used Logistic regression method to impute the categorical covariates with missing values. We found largely similar distributions of the observed and imputed variables. Increasing numbers of imputed datasets to 10 or 20 barely changed the estimates and their 95% confidence intervals of 5 imputed datasets.
