# Supplementary material for: Suicide attempt and death by suicide among parents of young individuals with cancer: A population-based study in Denmark and Sweden
Source: PLoS Med. 2024 Jan 16;21(1):e1004322. doi: 10.1371/journal.pmed.1004322 (PMC10791002; doi:10.1371/journal.pmed.1004322)
Supplement: Attachment — Submitted filename: Response_to_Reviewers.pdf [file pmed.1004322.s009.pdf]

Requests from the editors:

COMMENTS FROM THE EDITORS
GENERAL

Comment: Please respond to all editor and reviewer comments detailed below in full.

Response:
We have now responded to all comments of the editors and reviewers and made changes to the manuscript accordingly.

Comment: Please include line numbers starting at line 1 of the abstract and in continuous sequence thereafter and throughout.

Response:
We have now added line numbers throughout the manuscript as suggested.

Comment: Please ensure that the study is reported according to the STROBE guideline, and include the completed STROBE checklist as Supporting Information. Please add the following statement, or similar, to the Methods: "This study is reported as per the Strengthening the Reporting of Observational Studies in Epidemiology (STROBE) guideline (S1 Checklist)."
The STROBE guideline can be found here:
https://eur01.safelinks.protection.outlook.com/?url=http%3A%2F%2Fwww.equator-network.org%2Freporting-guidelines%2Fstrobe%2F&data=05%7C01%7Cqianwei.liu%40ki.se%7C2a505f0d39e8411da46108dbc42281c2%7Cbff7eef1cf4b4f32be3da1dda043c05d%7C0%7C0%7C638319424869850059%7CUnknown%7CTWFpbGZsb3d8eyJWIjoiMC4wLjAwMDAiLCJQIjoiV2luMzIiLCJBTiI6Ik1haWwiLCJXVCI6Mn0%3D%7C3000%7C%7C%7C&sdata=nz9ax6YqedQTzCziIp%2FiBEBt4StwjQ%2B0CjKNEXDxDPA%3D&reserved=0
When completing the checklist, please refer to section and paragraph numbers, rather than page or line numbers as these often change in the event of publication

Response:
We have now included a STROBE checklist as Supplementary Information and added the following statement to the Methods section (Pages 11, Lines 240-241):

"*This study is reported according to the Strengthening the Reporting of Observational Studies in Epidemiology (STROBE) guideline (**Supplementary S1 Checklist**).*"

Comment:* The editorial team agree that your manuscript addresses an important subject matter. However, the implications of your findings going forwards should be better discussed. *

Response:
Thank you for the encouraging comment. We have now discussed the implications of our findings in the Discussion section (Page 17, Lines 393-396):

"Our findings suggest the need of extended clinical awareness for the risk of suicide attempt among parents of children with cancer, primarily during the first years after cancer diagnosis.

Vigilance and interventions for severe psychological distress and psychiatric symptoms might be important and effective."

Comment: ** The editorial team agree with reviewer #3's concerns (please see below) regarding stratification according to sub-categories of childhood malignancy and require detailed justification for this. **

Response:
We performed separate analysis for CNS cancer, hematological malignancies, and other cancer types, because CNS cancer and hematological malignancies are the most common types of malignancies in early life, accounting for around 2/3 of all cancers diagnosed during childhood for instance. We have now clarified this in the Methods section (Page 8, Lines 153-156):

"*As cancers in central nervous system (CNS) and hematological malignancies are the most common types of cancer among young individuals, accounting for around two thirds of all childhood cancers for instance, in addition to any cancer, we classified cancers into CNS cancer, hematological malignancy, and other type of cancer.*"

Comment: *** We suggest tempering the claim of 'decreased' risks of suicide after 7 years and simply stating that increased risk is not maintained beyond 7 years ***

Response:
Thank you for the comment. We have now amended this, as suggested, throughout the manuscript.

In Abstract (Page 3, Lines 32-35):
"*The increased risk did not, however, maintain beyond seven years after cancer diagnosis (HR, 0.86; 95%CI: [0.75, 0.98]; p=0.03) and there was no altered risk of parental death by suicide any time after the child's cancer diagnosis.*"

In Results (Page 12, Lines 261-264):
"*We observed an increased risk of suicide attempt among the exposed parents during the first seven years after a child cancer diagnosis, but the increased risk did not maintain thereafter in either the unadjusted **(Supplementary S2A Fig)** or the adjusted **(Fig 1A)** model.*"

In Results (Page 13, Lines 285-288):
"*The lack of increased risk of suicide attempt beyond seven years after a child cancer diagnosis was, on the other hand, mainly attributable to fathers, older parents, as well as parents without a history of cancer or a family history of psychiatric disorder.*"

In Discussion (Page 14, Lines 313-315):
"*There was no increased risk of parental suicide attempt later during follow-up, and there was no altered risk of parental death by suicide any time after a child cancer diagnosis.*"

In Discussion (Page 15, Lines 340-343):
"In contrast to an increased risk of parental suicide attempt during the first years after a child cancer diagnosis, we observed no increased risk of parental suicide attempt later during

the follow-up and no altered risk of parental death by suicide any time after a child cancer diagnosis."

Comment: DATA AVAILABILITY STATEMENT
Thank you for including a detailed statement. Please provide a URL or email address for data enquiries to each data provider - Statistics Denmark, Swedish National Board of Health and Welfare, and Statistics Sweden. Please note that the contact cannot be a study author.

Response:
Thank you for the comment. We have now added URL of these in the DATA AVAILABILITY STATEMENT.

Comment: COMPETING INTERESTS
All authors must declare their relevant competing interests per the PLOS policy, which can be seen here:
https://eur01.safelinks.protection.outlook.com/?url=https%3A%2F%2Fjournals.plos.org%2Fplosmedicine%2Fs%2Fcompeting-interests&data=05%7C01%7Cqianwei.liu%40ki.se%7C2a505f0d39e8411da46108dbc42281c2%7Cbff7eef1cf4b4f32be3da1dda043c05d%7C0%7C0%7C638319424869850059%7CUnknown%7CTWFpbGZsb3d8eyJWIjoiMC4wLjAwMDAiLCJQIjoiV2luMzIiLCJBTiI6Ik1haWwiLCJXVCI6Mn0%3D%7C3000%7C%7C%7C&sdata=SaqLjITO%2FYp8hK8wLvv5oLX1l5qDVQZtSti%2B1Fjy%2FTc%3D&reserved=0
For authors with ties to industry, please indicate whether any of the interests has a financial stake in the results of the current study.

Response:
All authors have now declared their competing interests per the PLOS policy.

Comment: HIGHLIGHTS – page 2 – please remove this section.

Response:
We have now removed this section.

Comment: ABSTRACT
Please structure your abstract using the PLOS Medicine headings (Background, Methods and Findings, Conclusions).
Please combine the Methods and Findings sections into one section, "Methods and findings".

Response:
We have now modified the Abstract accordingly (Pages 2-3).

Comment: Abstract Background: Provide wider context of why the study is important. The final sentence should clearly state the study question.

Response:
We have now rephrased the Background section of the Abstract (Page 2, Lines 3-6):

"The psychological toll on parents of a child receiving a cancer diagnosis is known to be high, but there is a knowledge gap regarding suicidal behavior among these parents. The aim of

this study was to investigate the risk of suicide attempt and death by suicide in relation to having a child with cancer."

Comment: Abstract Methods and Findings:
Please ensure that all numbers presented in the abstract are present and identical to numbers presented in the main manuscript text.

Response:
We ensure that all numbers presented in the Abstract are present and identical to numbers presented in the main manuscript text.

Comment: Please provide a (brief) summary of aggregate demographic details of the study population – number of participants, sex, age (and range) and so on.

Response:
We have now added such information in the Abstract (Page 2, Lines 23-24):

"*The population comparison consisted of 106,005 exposed parents and 1,060,050 matched unexposed parents, with a median age of 56 at cohort entry and 46.9% male.*"

Comment: Please include the length of follow up (in mean, SD, and range) and main outcome measures.

Response:
We have now added such information in the Abstract.

In Pages 2-3, Lines 24-27:
"*During the median follow-up of 7.3 and 7.2 years, we observed 613 (incidence rate [IR], 58.8 per 100,000 person-years) and 5,888 (IR, 57.1 per 100,000 person-years) cases of first-onset suicide attempt among the exposed and unexposed parents, respectively.*"

In Page 2, Lines 12-15:
"*Suicide attempt was identified through the Patient Register and the Psychiatric Central Register in Denmark and the Patient Register in Sweden, whereas death by suicide was identified through the Danish Causes of Death Register and the Swedish Causes of Death Register.*"

Comment: Please quantify the main results with 95% CIs and p values. When reporting p values please report as p<0.001 and where higher the exact p value as p=0.002, for example. When reporting CIs please separate upper and lower bounds with commas as opposed to hyphens as the latter can be confused with reporting of negative values.
Suggest reporting statistical information as follows, '(HR, 1.15; 95%CI [1.03,1.28]; p </=’

Response:
We have now added p values alongside 95% CIs and reported 95% CIs and p values as required throughout the manuscript.

Comment: Please include any important dependent variables that are adjusted for in the analyses.

Response:
We have now added such information in the Abstract (Page 2, Lines 15-20):

"*In population comparison, we used Cox regression to estimate hazard ratios (HRs) and 95% confidence intervals (CIs) of suicide attempt and death by suicide associated with cancer diagnosis of a child, adjusting for sex, age, country of residence, calendar year, marital status, highest attained educational level, household income, history of cancer, history of psychiatric disorder, and family history of psychiatric disorder.*"

Comment: Please include the absolute risk(s) of relevant outcomes (including NNT or NNH where appropriate), not just relative risks or correlation coefficients. (example for absolute risks: PMID: 28399126).

Response:
We have now added incidence rates (IRs) in the Abstract (Pages 2-3, Lines 24-27):

"*During the median follow-up of 7.3 and 7.2 years, we observed 613 (incidence rate [IR], 58.8 per 100,000 person-years) and 5,888 (IR, 57.1 per 100,000 person-years) cases of first-onset suicide attempt among the exposed and unexposed parents, respectively.*"

Comment: In the last sentence of the Abstract Methods and Findings section, please describe the main limitation(s) of the study's methodology.

Response:
We have now described main limitation of the study in the Abstract (Page 3, Lines 35-36):

"*The main limitation of the study is the potential residual confounding by factors not shared between full siblings.*"

Comment: Abstract Conclusions:
Please address the study implications without overreaching what can be concluded from the data; the phrase "In this study, we observed ..." may be useful.

Response:
We have now rephrased the first sentence of Conclusion in the Abstract (Page 3, Lines 38-40):

"*In this study, we observed an increased risk of parental suicide attempt during the first years after a child's cancer diagnosis, especially when the child was diagnosed during childhood, or with an aggressive or fatal form of cancer.*"

Comment: Please interpret the study based on the results presented in the abstract, emphasizing what is new/what this adds/how this could influence change, without overstating your conclusions.

Response:
We have now added the following sentence in the Conclusion of the Abstract (Page 3, Lines 41-43):

"*Our findings suggest extended clinical awareness of suicide attempt among parents of children with cancer, especially during the first few years after cancer diagnosis.*"

Comment: Please avoid vague statements such as "these results have major implications for policy/clinical care". Mention only specific implications substantiated by the results.

Response:
We have now revised this sentence as shown in the above response.

Comment: Please ensure to avoid any assertions of primacy ("We report for the first time....")

Response:
We ensure that we avoided any assertions of primacy.

Comment: AUTHOR SUMMARY
At this stage, we ask that you include a short, non-technical Author Summary of your research to make findings accessible to a wide audience that includes both scientists and non-scientists. The authors summary should consist of 2-3 succinct bullet points under each of the following headings:

- Why Was This Study Done? Authors should reflect on what was known about the topic before the research was published and why the research was needed.
- What Did the Researchers Do and Find? Authors should briefly describe the study design that was used and the study's major findings. Do include the headline numbers from the study, such as the sample size and key findings.
- What Do These Findings Mean? Authors should reflect on the new knowledge generated by the research and the implications for practice, research, policy, or public health. Authors should also consider how the interpretation of the study's findings may be affected by the study limitations. In the final bullet point of 'What Do These Findings Mean?', please describe the main limitations of the study in non-technical language.

The Author Summary should immediately follow the Abstract in your revised manuscript. This text is subject to editorial change and should be distinct from the scientific abstract. Please see our author guidelines for more information: https://eur01.safelinks.protection.outlook.com/?url=https%3A%2F%2Fjournals.plos.org%2Fplosmedicine%2Fs%2Frevising-your-manuscript%23loc-author-summary&data=05%7C01%7Cqianwei.liu%40ki.se%7C2a505f0d39e8411da46108dbc42281c2%7Cbff7eef1cf4b4f32be3da1dda043c05d%7C0%7C0%7C638319424869850059%7CUnknown%7CTWFpbGZsb3d8eyJWIjoiMC4wLjAwMDAiLCJQIjoiV2luMzIiLCJBTiI6Ik1haWwiLCJXVCI6Mn0%3D%7C3000%7C%7C%7C&sdata=YIwVvASeZhhbzRUOFKBZFSKyoPytuPIMQpbV%2FR6mFLk%3D&reserved=0

Response:
We have now added a "AUTHOR SUMMMARY" section as guided (Page 4):

"***Author summary***
Why Was This Study Done?
Having a child with a cancer diagnosis is highly stressful for parents.

Little is known about risk of parental suicidal behavior in relation to cancer diagnosis of a child.

What Did the Researchers Do and Find?

We performed a binational population-based and sibling-controlled cohort study to investigate the association between cancer diagnosis of a child and the subsequent risk of suicide attempt or death by suicide among the parents.

We observed an increased risk of parental suicide attempt during the first years after a child's cancer diagnosis, especially when the child was diagnosed during childhood, or with an aggressive or fatal form of cancer, in both the population-based comparison and the sibling comparison.

What Do These Findings Mean?
Our findings call for clinical awareness of the risk of suicide attempt among parents of children with cancer, primarily within the first years after cancer diagnosis of the child.

Future studies are needed to examine the generalizability of our findings to other countries with different healthcare system, sociocultural context, and prevalence of cancer and suicidal behavior as Denmark and Sweden."

Comment: INTRODUCTION
The current introduction is rather brief. Please address past research and explain the need for and potential importance of your study. Indicate whether your study is novel and how you determined that. If there has been a systematic review of the evidence related to your study (or you have conducted one), please refer to and reference that review and indicate whether it supports the need for your study.

Response:
We have now modified the Introduction section to address past research and explain the need for and importance of our study, including indicating the novelty of our study. There is, to our best knowledge, currently no systematic review related to this research question.

In Introduction (Page 5, Lines 79-83):
"*The existing studies rendered largely conflicting findings, often based on relatively small sample size[5, 9, 10]. Most of these studies have had limited control for important confounding factors and focused on death by suicide alone, leaving the risk of suicide attempt unraveled[9, 10]. Moreover, none of these studies has explored the temporal pattern of suicidal behavior.*"

In Introduction (Page 5, Lines 85-90):
"*Considering the relatively low incidence of suicidal behavior, especially death by suicide, population-based studies with large sample size are needed to provide sufficient statistical power, especially for the analyses of temporal pattern in the risk of suicidal behavior and by cancer characteristics. Cross-validation of findings between populations as well as contrasting findings between suicide attempt and death by suicide in the same population are also of importance.*"

Comment: Please conclude the Introduction with a clear description of the study question or hypothesis, as in your current version.

Response:
We have now concluded the Introduction section with a clear description of the study question and hypothesis (Pages 5, Lines 90-96):

"*To this end, we took advantage of several national population and health registers in Denmark and Sweden and performed a population-based study to investigate the association between cancer diagnosis of a child and risk of parental suicide attempt and death by suicide, after adjusting for several potential confounders as well as controlling for familial confounding through a sibling comparison. We hypothesize that there is an altered risk of suicidal behavior among parents of children with cancer and that such risk alteration differs by time since cancer diagnosis and according to characteristics of the child's cancer.*"

Comment: METHODS and RESULTS
Please see comments below from reviewer #1 (statistical reviewer) regarding the approach to your analyses.
Please see reviewer #3 comments regarding sub-categorization of child hood malignancy which we require detailed justification for.

Response:
We have now responded to all comments from Reviewer #1 (please see our responses below) and the specific comment from Reviewer #3 (please see our response above).

Comment: Did your study have a prospective protocol or analysis plan? Please state this (either way) early in the Methods section.
a) If a prospective analysis plan (from your funding proposal, IRB or other ethics committee submission, study protocol, or other planning document written before analyzing the data) was used in designing the study, please include the relevant prospectively written document with your revised manuscript as a Supporting Information file to be published alongside your study, and cite it in the Methods section. A legend for this file should be included at the end of your manuscript.
b) If no such document exists, please make sure that the Methods section transparently describes when analyses were planned, and when/why any data-driven changes to analyses took place.
c) In either case, changes in the analysis—including those made in response to peer review comments—should be identified as such in the Methods section of the paper, with rationale.

Response:
Yes, our study has a prespecified analysis plan. We have now submitted this as Supplementary Information and stated so in the Methods section (Page 6, Lines 113-114):

"*The prespecified analysis plan of the present study is presented in the **Supplementary S1 Text**.*"

Comment: For all observational studies, we request that in the manuscript text, authors please indicate:
(1) the specific hypotheses you intended to test,
(2) the analytical methods by which you planned to test them,
(3) the analyses you actually performed, and
(4) when reported analyses differ from those that were planned, transparent explanations for differences that affect the reliability of the study's results. If a reported analysis was performed based on an interesting but unanticipated pattern in the data, please be clear that the analysis was data-driven.

Response:
We have now indicated or reported all above in the manuscript and declare hereby that no analysis presented in the manuscript is data-driven as described by the editors.

In Introduction (Page 5, Lines 90-96):

"*To this end, we took advantage of several national population and health registers in Denmark and Sweden and performed a population-based study to investigate the association between cancer diagnosis of a child and risk of parental suicide attempt and death by suicide, after adjusting for several potential confounders as well as controlling for familial confounding through a sibling comparison. We hypothesize that there is an altered risk of suicidal behavior among parents of children with cancer and that such risk alteration differs by time since cancer diagnosis and according to characteristics of the child's cancer.*"

In Methods (Page 9, Lines 191-195):

"*We calculated the incidence rates (IRs) of suicide attempt and death by suicide among the exposed and unexposed parents, through dividing number of cases by accumulated person-years at risk. We visualized the time-varying associations of child cancer diagnosis with the risk of parental suicide attempt and death by suicide using flexible parametric survival models, in both the population and sibling comparisons.*"

In Methods (Pages 10, Lines 198-207):

"*In the population comparison, the analyses were fitted in two models. In the first model, we performed the analyses without any adjustment. In the second model, we adjusted for sex, age at the index date, country of residence, calendar year of the index date, marital status, highest attained educational level, household income, history of cancer, history of psychiatric disorder, and family history of psychiatric disorder. In the sibling comparison, we also performed the analyses using two models. In the unadjusted model, the analyses were stratified by family identifier (mother's and father's identification numbers). In the adjusted model, we additionally adjusted for the same variables as in population comparison except family history of psychiatric disorder.*"

In Methods (Page 10, Lines 209-213):

"We then moved on to analyze the effect of parental and child cancer characteristics on the association of interest. In these analyses, we used Cox regression to estimate the average hazard ratio (HR) and 95% confidence interval (CI) of suicide attempt and death by suicide in relation to a child cancer diagnosis, with time since the index date as the underlying time scale and the same adjustment as described in flexible parametric survival models."

Comment: Please report the number of patients and dates of recruitment, and account for all methods used in your study.

Response:
We have now reported all information in the manuscript as required.

In Methods (Pages 6-7, Lines 116-128):

"*We first identified all parents that had a child diagnosed with cancer during 1978-2016 in Denmark (N=52,027) or 1973-2014 in Sweden (N=59,428)* ***(Supplementary S1 Fig)****. If a parent had more than one child with a cancer diagnosis, the date of cancer diagnosis of the first child was regarded as the index exposure. As we aimed to study newly diagnosed cancer in a child and the first-onset parental suicide attempt or death by suicide, parents that had a child diagnosed with cancer (N=283 in Denmark and N=149 in Sweden) or had a suicide attempt (N=998 in Denmark and N=1,169 in Sweden) before the study period were excluded from the main analysis. To ensure relatively complete ascertainment of familial links from the Danish Civil Registration System and Swedish Multi-Generation Register, we also excluded parents born before 1936 in Denmark (N=1,914) or before 1932 in Sweden (N=944). Finally, three parents in Sweden with conflicting information on their children (i.e., a child died or emigrated before date of birth) were also excluded, leaving 106,005 parents (N=48,842 in Denmark and N=57,163 in Sweden) in analysis.*"

In Methods (Page 7, Lines 137-140):

"*We identified 64,446 unexposed full siblings (of 40,218 exposed parents), who had no history of suicide attempt and at least one child alive with no diagnosed cancer, before the index date of their exposed full sibling.*"

In Methods (Pages 7-8, Lines 143-147):

"*We then followed all study participants from the index date, until death, emigration, an incidence of suicide attempt or death by suicide, or the end of follow-up (December 31st, 2016 in Denmark and December 31st, 2014 in Sweden), whichever came first. Follow-up of the unexposed parents and the unexposed siblings was additionally censored if they had a child diagnosed with cancer during follow-up.*"

In Methods (Page 9, Lines 191-195):

"*We calculated the incidence rates (IRs) of suicide attempt and death by suicide among the exposed and unexposed parents, through dividing number of cases by accumulated person-years at risk. We visualized the time-varying associations of child cancer diagnosis with the risk of parental suicide attempt and death by suicide using flexible parametric survival models, in both the population and sibling comparisons."*

In Methods (Page 10, Lines 198-207):

"*In the population comparison, the analyses were fitted in two models. In the first model, we performed the analyses without any adjustment. In the second model, we adjusted for sex, age at the index date, country of residence, calendar year of the index date, marital status, highest attained educational level, household income, history of cancer, history of psychiatric disorder, and family history of psychiatric disorder. In the sibling comparison, we also performed the analyses using two models. In the unadjusted model, the analyses were stratified by family identifier (mother's and father's identification numbers). In the adjusted model, we additionally adjusted for the same variables as in population comparison except family history of psychiatric disorder.*"

In Methods (Page 10, Lines 209-213):

“We then moved on to analyze the effect of parental and child cancer characteristics on the association of interest. In these analyses, we used Cox regression to estimate the average hazard ratio (HR) and 95% confidence interval (CI) of suicide attempt and death by suicide in relation to a child cancer diagnosis, with time since the index date as the underlying time scale and the same adjustment as described in flexible parametric survival models.”

Comment: As for the abstract, please quantify the main results with 95% CIs and p values. When reporting p values please report as p<0.001 and where higher the exact p value as p=0.002, for example. When reporting CIs please separate upper and lower bounds with commas as opposed to hyphens as the latter can be confused with reporting of negative values. Suggest reporting statistical information as follows, ‘(HR, 1.15; 95%CI [1.03,1.28]; p </=’ When a p value is given, please specify the statistical test used to determine it.

Response:
We have now added p values alongside 95% CIs and reported 95% CIs and p values as required throughout the manuscript. We have also specified the test used to determine p values in the Methods section (Page 11, Lines 242-243):

“*Statistical significance was indicated using P < 0.05 and Wald test was used to determine P values.*”

Comment: Page 6 – ‘cancer child’ please refrain from using this term, suggest ‘child with a cancer diagnosis’ or similar. Please check and amend throughout all sections of the manuscript including the supporting files where relevant.

Response:
Thank you for the great suggestion. We have now replaced this term with “child with cancer” or “children with cancer” throughout the manuscript.

Comment: Page 11 – please define ‘IR’ at first use for the reader – apologies if I have missed it.

Response:
Thank you for the comment. We have now clarified this in the Methods section (Page 9, Lines 191-193):

“*We calculated the incidence rates (IRs) of suicide attempt and death by suicide among the exposed and unexposed parents, through dividing number of cases by accumulated person-years at risk.*”

Please define the length of follow up (eg, in mean, SD, and range).

Response:
We have now stated the median length of follow-up in the Results section (Page 12, Lines 258-259):

“*In the population comparison, the median follow-up time was 7.3 years for the exposed and 7.2 years for the unexposed parents.*”

Comment: At the beginning of your results section (PDF page 11) please include a brief summary of the aggregate demographic details of the study population – number of participants, sex, age (and range) and so on.

Response:
We have now added such information in the Results section (Page 11, Lines 251-252):

"*The population comparison consisted of 106,005 exposed parents and 1,060,050 matched unexposed parents, with a median age of 56 at cohort entry and 46.9% male.*"

Comment: TABLES
Table 2
I found this table a little confusing. It wasn't entirely clear (without referring to the text) how participant numbers apply to the results presented. Please ensure that the table can be interpreted by the reader without the need to refer to the text. Please indicate what the different results in bold refer to.

Response: Thank you for the comment. We have now adapted table 2 to make it clearer. We have also indicated the meaning of the results in bold.

Table 2. Incidence rate (IR, per 100,000 person-years) and hazard ratio (HR) with 95% confidence interval (CI) of suicide attempt following a child cancer diagnosis – analysis of the population comparison

Characteristics	Parents of cancer-free children		Parents of children with cancer							
	Entire follow-up		Entire follow-up		First 7 years of follow-up			>7 years of follow-up		
	No. of cases	IR	No. of cases	IR	Unadjusted HR (95% CI)[a]; *P* value	Adjusted HR (95% CI)[b] ; *P* value	*P* for interaction	Unadjusted HR (95% CI)[a]; *P* value	Adjusted HR (95% CI)[b]; *P* value	*P* for interaction
**Overall**	5,888	57.1	613	58.8	**1.17 (1.11, 1.23); P<0.001**	**1.15 (1.03, 1.28);**		**0.87 (0.82, 0.92); P<0.001**	**0.86 (0.75, 0.98); P=0.03**	
**Sex**							0.15			0.17
Male	2,531	52.4	235	48.1	1.04 (0.97, 1.13); P=0.27	1.04 (0.88, 1.24);		**0.76 (0.69, 0.84); P<0.001**	**0.77 (0.62, 0.95); P=0.02**	
Female	3,357	61.2	378	68.2	**1.27 (1.19, 1.35); P<0.001**	**1.23 (1.07, 1.41);**		0.95 (0.88, 1.02); P=0.15	0.93 (0.79, 1.10); P=0.40	
**Age at cohort entry,**							0.04			0.11
<40	2,765	82.1	297	87.1	**1.25 (1.15, 1.35); P<0.001**	**1.27 (1.06, 1.52);** [illegible]		0.95 (0.88, 1.02); P=0.15	0.96 (0.81, 1.12); P=0.58	
40-60	2,583	47.9	273	50.2	**1.22 (1.14, 1.30); P<0.001**	**1.18 (1.02, 1.36);**		**0.75 (0.67, 0.83); P<0.001**	**0.73 (0.57, 0.93); P=0.01**	
>60	540	34.6	43	27.3	**0.80 (0.70, 0.94); P=0.005**	0.79 (0.56, 1.09);		**0.54 (0.35, 0.85); P=0.008**	0.53 (0.19, 1.45); P=0.15	
**Calendar year at**							0.18			0.73
1973-1989	1,579	68.9	166	71.5	**1.34 (1.19, 1.50); P<0.001**	**1.32 (1.02, 1.71);**		**0.91 (0.83, 0.99); P=0.04**	0.90 (0.73, 1.11); P=0.32	
1990-1999	1,860	61.4	190	62.2	**1.32 (1.20, 1.44); P<0.001**	**1.31 (1.06, 1.61);** [illegible]		**0.80 (0.73, 0.89); P<0.001**	0.80 (0.65, 1.00); **P=0.05**	
2000-2009	1,893	51.1	199	53.1	**1.08 (1.00, 1.17); P=0.04**	1.06 (0.89, 1.25);		0.92 (0.80, 1.05); P=0.22	0.91 (0.67, 1.23); P=0.54	
2010-2016	556	43.1	58	44.6	1.04 (0.92, 1.18); P=0.51	1.01 (0.77, 1.32);		-	-	
**Country of residence**							0.43			0.36

Sweden	3,101	54.6	320	55.9	**1.21 (1.14, 1.30); P<0.001**	**1.19 (1.03, 1.38); [illegible]**		**0.82 (0.75, 0.89); P<0.001**	**0.82 (0.68, 0.98); P=0.03**	
Denmark	2,787	60.1	293	62.4	**1.12 (1.05, 1.20); P=0.001**	1.10 (0.94, 1.28); [illegible]		0.93 (0.85, 1.01): P=0.10	0.91 (0.75, 1.11); P=0.35	
**Educational**							0.79			0.66
0-9	2,252	81.3	249	82.6	**1.11 (1.03, 1.20); P=0.008**	1.12 (0.94, 1.33);		**1.11 (1.03, 1.20); P=0.008**	0.92 (0.75, 1.12); P=0.40	
10-14	2,904	52.3	294	52.4	**1.17 (1.09, 1.25); P<0.001**	1.16 (0.99, 1.34);		**0.80 (0.73, 0.87); P<0.001**	**0.80 (0.66, 0.98); P=0.03**	
≥15	582	31.5	58	35.0	**1.22 (1.05, 1.41); P=0.008**	1.26 (0.91, 1.76);		0.94 (0.77, 1.16); P=0.57	0.97 (0.61, 1.55); P=0.89	
**Household income**							0.76			0.88
Below the lowest tertile	1,897	89.7	200	96.2	**1.13 (1.05, 1.22); P=0.001**	1.10 (0.93, 1.30);		0.94 (0.83, 1.06); P=0.29	0.90 (0.68, 1.18); P=0.44	
Between the lowest and highest tertile	1,745	65.6	192	68.6	**1.19 (1.09, 1.30); P<0.001**	1.17 (0.96, 1.43); P=0.12		**0.87 (0.80, 0.96); P=0.005**	0.87 (0.70, 1.08); P=0.21	
Above the highest tertile	1,880	37.8	186	37.5	**1.20 (1.10, 1.31); P<0.001**	1.19 (0.98, 1.44);		**0.82 (0.74, 0.91); P<0.001**	0.83 (0.66, 1.04); P=0.11	
**Marital status**							0.34			0.88
Single, widowed or	2,685	96.8	261	100.3	**1.13 (1.05, 1.21); P<0.001**	1.09 (0.93, 1.27);		**0.87 (0.80, 0.95); P=0.003**	0.85 (0.69, 1.05); P=0.13	
Married	2,722	42.9	303	46.4	**1.25 (1.16, 1.33); P<0.001**	**1.21 (1.04, 1.40);**		**0.89 (0.82, 0.97); P=0.005**	0.87 (0.72, 1.04); P=0.14	
**History of cancer**							0.28			0.42
Yes	175	50.8	22	50.0	0.90 (0.71, 1.13); P=0.37	0.89 (0.53, 1.50);		1.30 (0.88, 1.92); P=0.19	1.17 (0.49, 1.80); P=0.72	
No	5,731	57.7	591	59.2	**1.18 (1.13, 1.24); P<0.001**	**1.16 (1.04, 1.30);**		**0.86 (0.81, 0.92); P<0.001**	**0.86 (0.75, 0.98); P=0.02**	
**History of psychiatric disorder**							0.27			0.87
Yes	1,829	317.0	193	317.5	1.07 (0.99, 1.16); P=0.09	1.07 (0.90, 1.27);		**0.84 (0.74, 0.96); P=0.01**	0.85 (0.63, 1.14); P=0.27	
No	4,059	41.7	420	42.8	**1.21 (1.14, 1.28); P<0.001**	**1.20 (1.05, 1.37);**		**0.87 (0.81, 0.93); P<0.001**	0.86 (0.74, 1.00); P=0.06	

**Family history of psychiatric [illegible]**							0.15			0.38
Yes	1,143	82.9	113	55.3	1.00 (0.90, 1.12); P=1.00	0.98 (0.76, 1.25);		0.98 (0.86, 1.13); P=0.82	0.98 (0.72, 1.34); P=0.90	
No	4,745	53.1	500	82.0	1.22 (1.15, 1.28)**; P<0.001**	**1.19 (1.06, 1.34);**		**0.85 (0.79, 0.91); P<0.001**	**0.84 (0.72, 0.97); P=0.02**	

[a] Without any adjustment. Time since cohort entry was used as the underlying time scale. The analyses were performed on five imputed datasets and HRs were obtained from combination of each dataset using Rubin's rule.

[b]Adjusted for sex, age at cohort entry, country of residence, calendar year at cohort entry, marital status, the highest attained educational level, household income, history of cancer, history of psychiatric disorder, and family history of psychiatric disorder. Time since cohort entry was used as the underlying time scale. The analyses were performed on five imputed datasets and HRs were obtained from combination of each dataset using Rubin's rule.

Bold confidence intervals and p values refer to statistically significant results.

Comment: Please separate upper and lower bounds of CIs with commas as opposed to hyphens as the later can be confused with reporting of negative values.

Response:
We have now modified this throughout the manuscript.

Comment: Thank you for indicating that your results are adjusted. To help facilitate transparent data reporting please also include unadjusted analyses for comparison.

Response:
We have now added unadjusted results in Table 2, please see our response to your comment about Table 2 above.

Comment: Table 3
Where reporting 95% CIs please also report p values. If not reporting p values for the purpose of transparent data reporting, please clearly state the reasons why not.

Response:
We have now reported p values in Table 3.

Table 3 Hazard ratio (HR) with 95% confidence interval (CI) of suicide attempt during the first seven years after a child cancer diagnosis - subgroup analysis of population comparison by cancer characteristics and calendar period

Cancer characteristics	HR (95% CI)[a]	P value
**Age at cancer diagnosis**		
≤18 years	**1.25 (1.08, 1.46)**	**0.004**
>18 years	1.07 (0.92, 1.24)	0.36
**Cancer type**		
Central nervous system (CNS) tumor	1.18 (0.91, 1.54)	0.21
Hematological malignancy	**1.26 (1.02, 1.55)**	**0.03**
Other cancer types	1.10 (0.96, 1.26)	0.16
**Cancer aggressiveness[b]**		
Low	1.10 (0.89, 1.37)	0.37
Medium	1.13 (1.00, 1.28)	0.05
High	**1.60 (1.05, 2.43)**	**0.03**
**Survival status**		
Surviving	1.07 (0.95, 1.21)	0.25
Deceased	**1.63 (1.29, 2.06)**	**<0.001**

[a]Cox models were adjusted for sex, age at cohort entry, country of residence, calendar year at cohort entry, marital status, the highest attained educational level, household income, history of cancer, history of psychiatric disorder, and family history of psychiatric disorder. Time since cohort entry was used as the underlying time scale. The analyses of Cox models were performed on five imputed datasets and HRs were obtained from combination of each dataset using Rubin's rule.
[b]Cancer aggressiveness was defined according to 5-year survival rate of each cancer type (https://www-dep.iarc.fr/nordcan/english/frame.asp.). Low aggressiveness of cancer type includes breast cancer, prostate cancer, non-melanoma skin cancer, melanoma, corpus uteri cancer and thyroid cancer. High aggressiveness of cancer type includes lung cancer, oesophagus cancer, liver cancer, pancreatic cancer, ovary cancer and unknown/illed-defined cancer. Medium aggressiveness of cancer type includes other cancer types not included in low aggressiveness and high aggressiveness.
Bold confidence intervals and p values refer to statistically significant results.

Comment: FIGURES
Figure 1 A-D – thank you for indicating your analyses are adjusted and detailing the factors you have adjusted for. To help facilitate transparent data reporting please also include the unadjusted analyses for comparison. In the caption please clearly define 'df' for the reader (degrees of freedom?).
Please clearly define the meaning of the different colour lines on the graphs.
Suggest perhaps swapping these colours as the reader is drawn to the colour first but the coloured line this is not the primary feature (the black line should be). Suggest also making the line at 1 dashed to further minimize its prominence.

Response:
Thank you for the suggestions. We have now modified Figure 1, including clarifying the caption, as suggested. We have also performed unadjusted analyses for Figure 1 and present these new results in Supplementary S2 Fig. We describe the corresponding methods and results in the Methods and Results sections, as following:

Fig 1 Adjusted hazard ratio (HR) with 95% confidence interval (CI) of suicide attempt and death by suicide in relation to cancer diagnosis of a child, analyses of population or sibling comparison):

A. Suicide attempt in population comparison

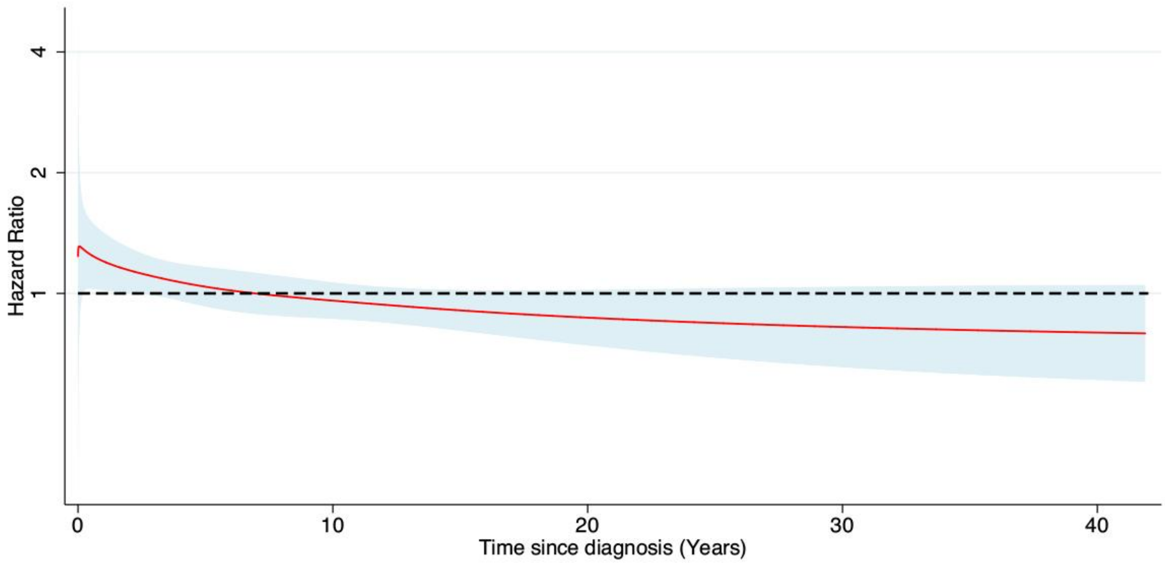

B. Suicide attempt in sibling comparison

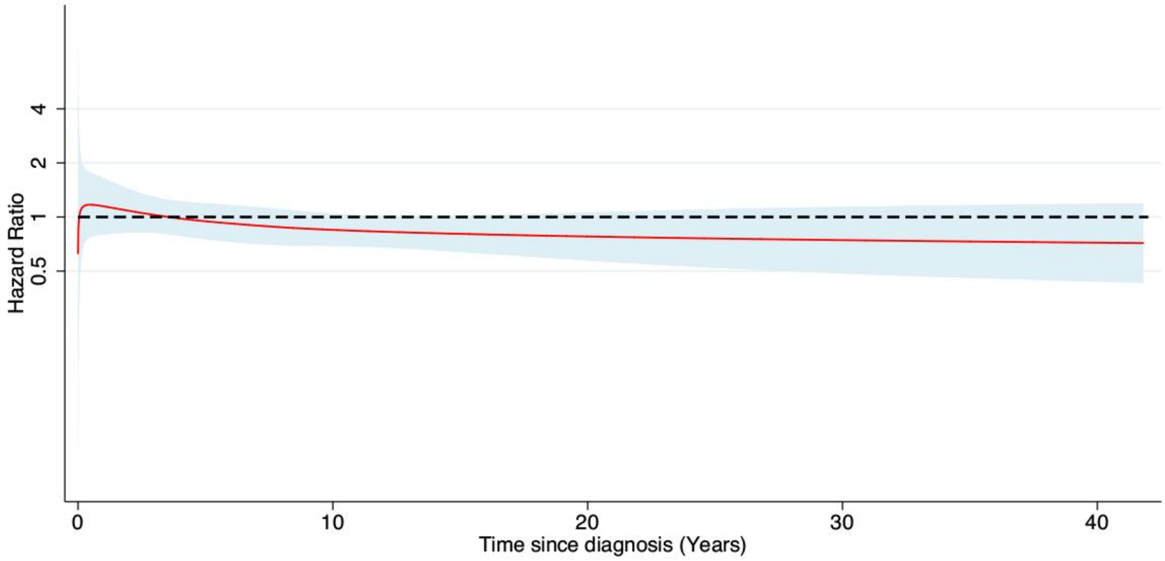

C. Death by suicide in population comparison

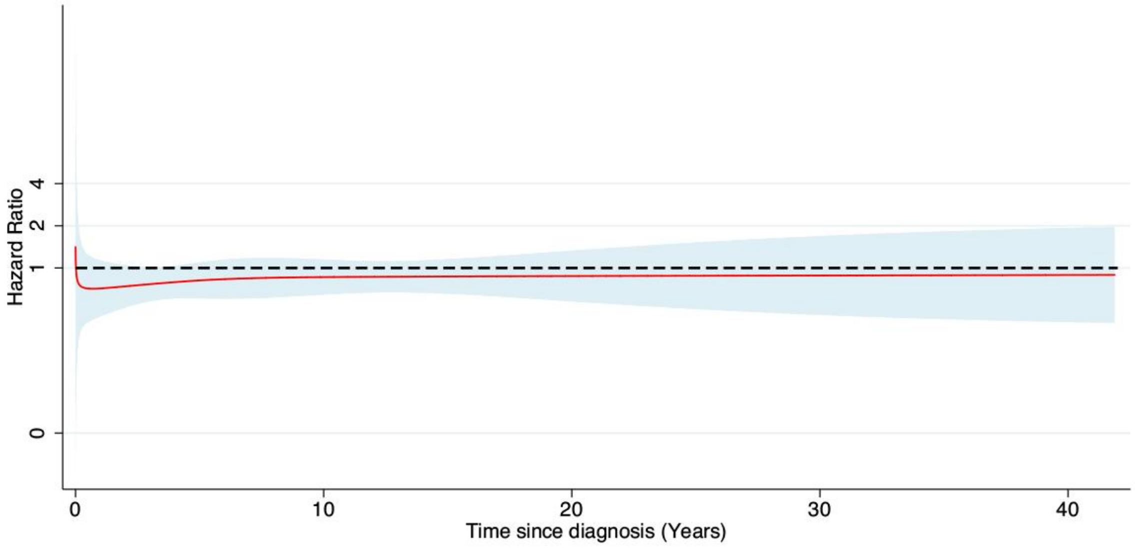

D. Death by suicide in sibling comparison

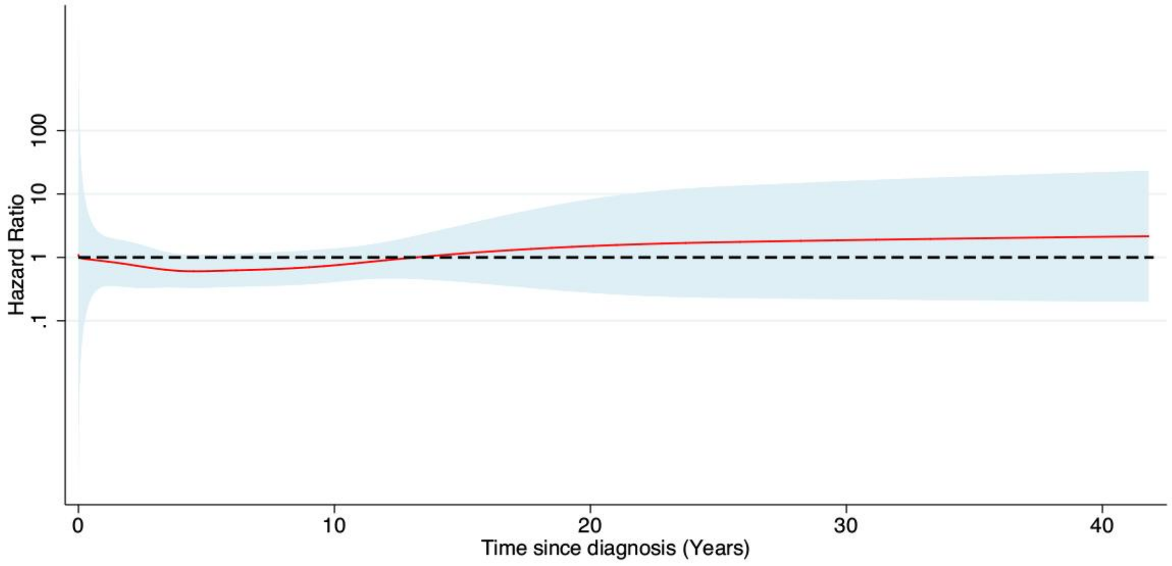

Unadjusted figures (Supplementary S2 Fig Unadjusted hazard ratio (HR) with 95% confidence interval (CI) of suicide attempt and death by suicide in relation to cancer diagnosis of a child, analyses of population or sibling comparison):

A. Suicide attempt in population comparison

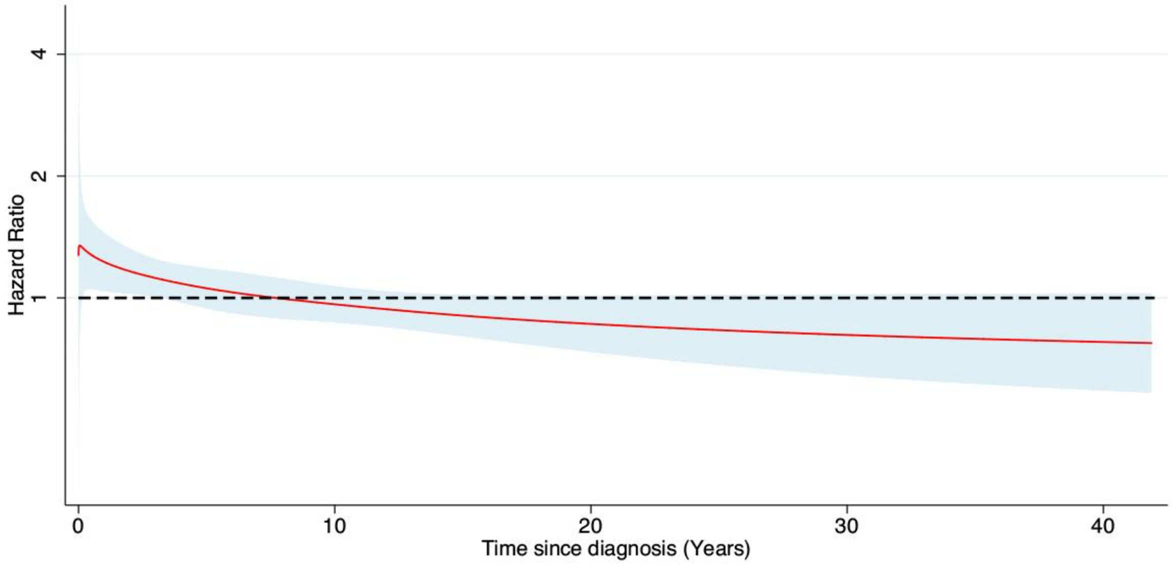

B. Suicide attempt in sibling comparison

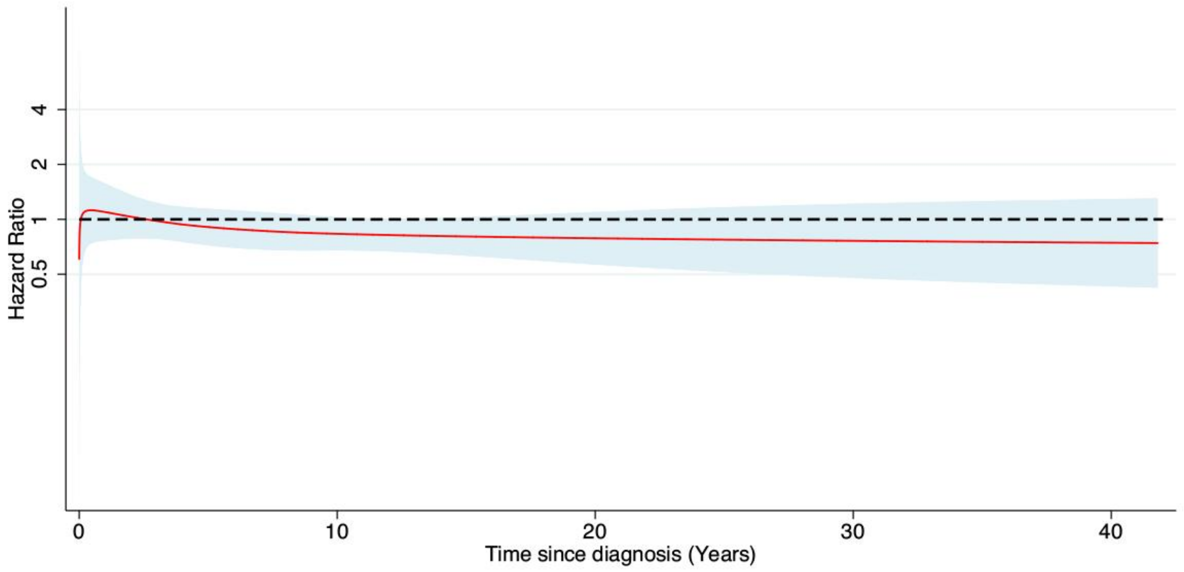

C. Death by suicide in population comparison

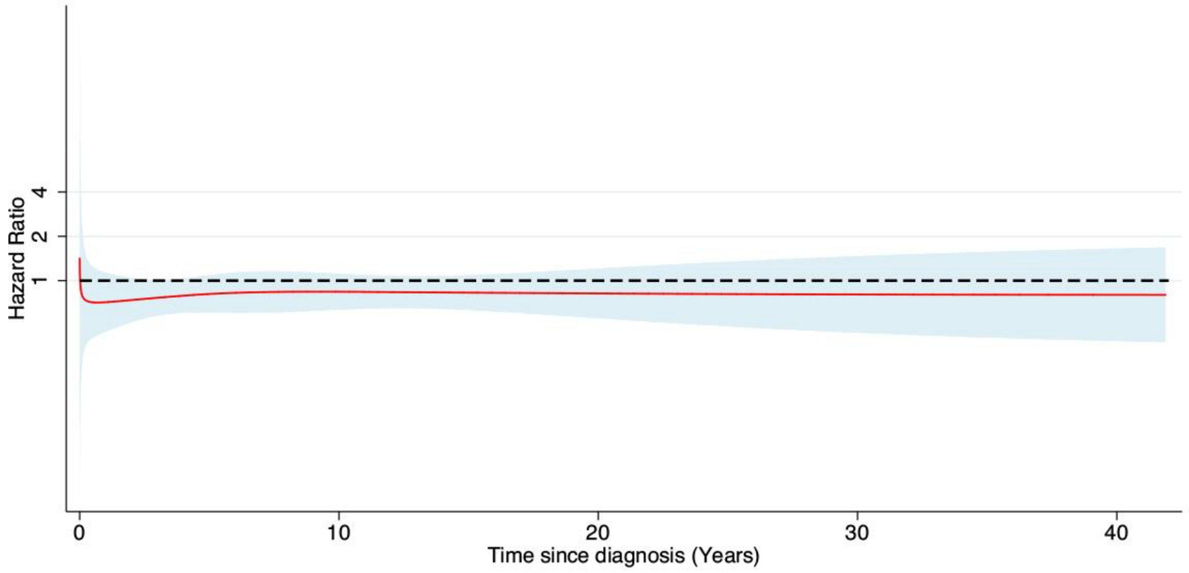

D. Death by suicide in sibling comparison

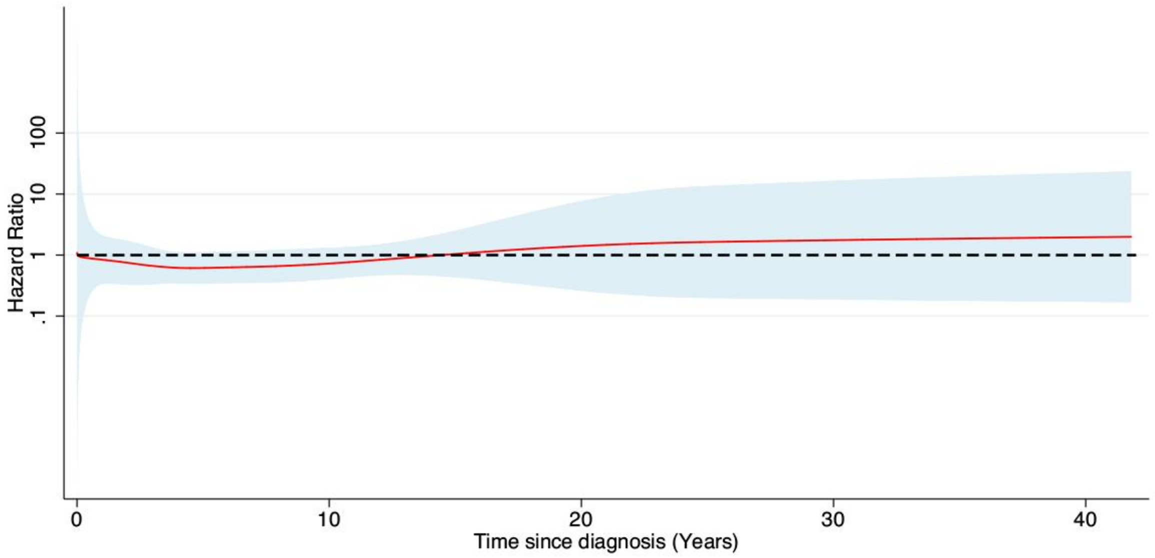

In Methods (Page 10, Lines 198-207):

"*In the population comparison, the analyses were fitted in two models. In the first model, we performed the analyses without any adjustment. In the second model, we adjusted for sex, age at the index date, country of residence, calendar year of the index date, marital status, highest attained educational level, household income, history of cancer, history of psychiatric disorder, and family history of psychiatric disorder. In the sibling comparison, we also performed the analyses using two models. In the unadjusted model, the analyses were stratified by family identifier (mother's and father's identification numbers). In the adjusted model, we additionally adjusted for the same variables as in population comparison except family history of psychiatric disorder.*"

In Results (Page 12, Lines 261-264):

"*We observed an increased risk of suicide attempt among the exposed parents during the first seven years after a child cancer diagnosis, but the increased risk did not maintain thereafter in either the unadjusted* ***(Supplementary S2A Fig)*** *or the adjusted* ***(Fig 1A)*** *model.*"

Comment: DISCUSSION
Please present and organize the Discussion as follows: a short, clear summary of the article's findings; what the study adds to existing research and where and why the results may differ from previous research; strengths and limitations of the study; implications and next steps for research, clinical practice, and/or public policy; one-paragraph conclusion. Please avoid the use of sub-headings such that the discussion reads as continuous prose.

Response:
We have now organized the Discussion section as suggested and removed sub-headings.

Comment: Please remove the funding and disclosure statements from the end of the main manuscript and include only in the masncuript submission form. In the event of publication they will be compiled as metadata..

Response:
We have now removed the funding and disclosure statements.

Comment: REFERENCES
For in-text reference callouts please place citations is square brackets and preceding punctuation. For example, [1,4,6].
In the bibliography please list up to but no more than 6 author names followed by et al in the event that more than 6 authors contribute to an individual study. Please ensure that journal name abbreviations are those listed in National Center for Biotechnology Information (NCBI) databases.
Please see our website for other reference guidelines
https://eur01.safelinks.protection.outlook.com/?url=https%3A%2F%2Fjournals.plos.org%2Fplosmedicine%2Fs%2Fsubmission-guidelines%23loc-references&data=05%7C01%7Cqianwei.liu%40ki.se%7C2a505f0d39e8411da46108dbc42281c2%7Cbff7eef1cf4b4f32be3da1dda043c05d%7C0%7C0%7C638319424869850059%7CUnknown%7CTWFpbGZsb3d8eyJWIjoiMC4wLjAwMDAiLCJQIjoiV2luMzIiLCJBTiI6Ik1haWwiLCJXVCI6Mn0%3D%7C3000%7C%7C%7C&sdata=6L8AFi3HyIrIcz8MrLBeMFlTUsRGPM8mUb1jUBvngYg%3D&reserved=0

Response:
We have now adapted reference to the guided style.

Comment: SUPPORTING INFORMATION
Please cite your Supporting Information as outlined here:
https://eur01.safelinks.protection.outlook.com/?url=https%3A%2F%2Fjournals.plos.org%2Fplosmedicine%2Fs%2Fsupporting-information&data=05%7C01%7Cqianwei.liu%40ki.se%7C2a505f0d39e8411da46108dbc42281c2%7Cbff7eef1cf4b4f32be3da1dda043c05d%7C0%7C0%7C638319424869850059%7CUnknown%7CTWFpbGZsb3d8eyJWIjoiMC4wLjAwMDAiLCJQIjoiV2luMzIiLCJBTiI6Ik1haWwiLCJXVCI6Mn0%3D%7C3000%7C%7C%7C&sdata=rZntXQBdWxCN3sEFL%2FbImzXmzdzxR8q8nEWgRzqJ%2F5s%3D&reserved=0
In the published article, supporting information files are accessed only through a hyperlink attached to the captions.

Response:
We have now cited Supporting Information as suggested.

Comment: For this reason, you must list captions at the end of your manuscript file. You may include a caption within the supporting information file itself, as long as that caption is also provided in the manuscript file. Do not submit a separate caption file.

Response:
We have now listed captions as guided.

Comment: As above, please include the completed STROBE checklist as Supporting Information. Please ensure to refer to section and paragraph numbers as opposed to page or line numbers as these often change in the event of publication.

Response:

We have now submitted a STROBE checklist as Supplementary Information and talked about this list in the Methods section (Page 11, Lines 240-241):

"*This study is reported according to the Strengthening the Reporting of Observational Studies in Epidemiology (STROBE) guideline (**Supplementary S1 Checklist**).*"

Comment: SUPPLEMENTARY FIGURES
As for the main manuscript suggest revising the graphs.

Response:
We have now revised supplementary figures as guided (Please see the response to the comment on figures above).

Comment: COMMENTS FROM THE ACADEMIC EDITOR
I looked through the paper in detail and agree that it seems important and well-conducted.

Response:
Thank you for the encouraging comment.

Comment: My main observation was that the difference between the findings for suicide attempt and death by suicide (preferred terminology to 'completed suicide') might be explained by ascertainment bias. Many suicide attempts will not come to the attention of the health service and be picked up in the registry. I thought it was plausible that parents of a child with cancer would be having more contact with health services (albeit not for themselves) and that this could facilitate their presentation to services with a suicide attempt (e.g. family support may mean the parent has the opportunity to disclose). That said there is consistency of findings across the different analyses that support their veracity.

Response:
Thank you for the comment. We understand the concern of the editor regarding potential better ascertainment of suicide attempt due to greater access of healthcare service among parents of children with cancer. However, on the other hand, we may also argue that parents of children with cancer might have lower chance of presenting themselves to health care in case of ill health, due to psychological stress and the high demand of parenting a critically ill child. Regardless, we agree with the editor that the consistency of findings across the different analyses supports the veracity of our findings. We have now added discussions on this in the Discussion section (Page 17, Lines 372-376):

"*Third, there is a concern of surveillance bias, assuming parents of children with cancer might have a greater access to health care, leading to a greater possibility of being recorded for suicide attempt in the healthcare system, or, alternatively, a lower chance of presenting themselves to health care in case of ill health, due to psychological distress or the demanding parenting of a child with cancer.*"

Comment: Some minor other points:
- The term 'child' is accurate but is somehow misleading. It is really being used in the term of offspring/son/daughter and not someone who is a 'child' i.e. below the age of 18 years. This becomes apparent as you read the paper but is not what I expected from the title.

Response:
Thank you for the comment. We have now adapted the title to "***Suicide attempt and death by suicide among parents of young individuals with cancer: a population-based study in Denmark and Sweden***"

Comment: - In the methods the term ‘a cancer child’ needs to be amended e.g. to ‘a child with cancer’.

Response:
We have now replaced this expression by "child with cancer" or "children with cancer" throughout the manuscript.

Comment: - In the discussions, the increased risk of suicide attempts in mothers is explained as "likely because mothers tend to have a stronger emotional link with their children". That seems highly speculative and potentially offensive so would need to be well-supported by citations evidence (that I don’t think exists).

Response:
Thank you for the comment. We agree and have now modified this sentence in the Discussion section (Page 15, Lines 332-334):

"The former is likely because females might be more susceptible to psychological distress than males[25, 26]"

Comments from the reviewers:

*** Reviewer #1:

Comment: This is a well-conducted population-based study in Denmark and Sweden on suicidal attempt and completed suicide among parents of children with cancer. The study design, datasets, statistical methods and analyses, and presentation (tables and figures) and interpretation of the results are mostly adequate.

Response:
Thank you for the very encouraging comments!

However, there are still a few statistical issues needing attention.

Response:
Thank you – we have now addressed the issues raised one-by-one below and made changes to the manuscript accordingly.

Comment： 1) On page 11, it's said "We observed an increased risk of suicidal attempt among the exposed parents during the first seven years after a child cancer diagnosis but a decreased risk thereafter (Figure 1A)". However, Figure 1A shows only dereasing HRs - it's one way traffic. Where does this increase and then decrease come from? The 7 years cut off is a bit arbitrary and perhaps more solid and robust evidence are needed to support this. On the other hand, as we can see the proportional hazard assumption doesn't hold in this study. A time-dependent survival analysis could be considered instead of the Cox model, although stratified analyses may solve the issue in some degree..

Response:
Thank you for the comment. We used "increased risk" to indicate that the HR of suicide attempt in relation to cancer diagnosis of a child was above 1, whereas used "decreased risk" to indicate that the HR was below 1. As we calculated the HR at each year of follow-up, we found that HR became below 1 after seven years of follow-up. Regardless, we agree with the reviewer that the 7-year cut-off is potentially study specific, and have now toned down about this cut-off throughout the manuscript.

In the Abstract section (Page 3, Lines 38-40):

"*In this study, we observed an increased risk of parental suicide attempt during the first years after a child's cancer diagnosis, especially when the child was diagnosed during childhood, or with an aggressive or fatal form of cancer.*"

In the Discussion section (Page 14, Lines 311-313):

"*In this binational cohort study, we observed an increased risk of parental suicide attempt during the first years after a child cancer diagnosis, particularly when the child was diagnosed at 18 or younger or when the child was diagnosed with an aggressive or fatal cancer.*"

In the Discussion section (Page 17, Lines 389-391):

"*In conclusion, there was an increased risk of parental suicide attempt during the first years after the diagnosis of a child cancer, particularly when the child was diagnosed at age 18 or below, or when the child was diagnosed with a cancer of poor prognosis, but not thereafter. There was no altered risk of parental death by suicide any time after a child cancer diagnosis.*"

We agree with the reviewer that it is important to show the potentially time-varying association between child cancer diagnosis and parental suicidal behavior, and this is precisely one of our primary aims in the study. Therefore, before using Cox model to show average HR during different periods of follow-up, we performed flexible parametric models to show the time-varying HR of parental suicidal behavior in relation to a child cancer diagnosis. We have now clarified this in the manuscript.

In the Introduction section (Page 5, Lines 85-88):

"*Considering the relatively low incidence of suicidal behavior, especially death by suicide, population-based studies with large sample size are needed to provide sufficient statistical power, especially for the analyses of temporal pattern in the risk of suicidal behavior and by cancer characteristics.*"

In the Methods section (Page 9, Lines 193-195):

"*We visualized the time-varying associations of child cancer diagnosis with the risk of parental suicide attempt and death by suicide using flexible parametric survival models, in both the population and sibling comparisons.*"

Comment: 2) Competing risk. As the outcomes of the Cox model are attempted suicide and completed suicide rather than all-cause mortality, there exists a competing risk from death on these suicide outcome. Can authors show the percentages of death (all cause mortality) in these cohorts? Fine and Gray competing risk regression model could be considered in this situation.

Response:
Thank you for the comment. We understand the concern of the reviewer on competing risk, as parents of children with cancer might have a higher mortality than others. If there were indeed a positive association between having a child with cancer and parental risk of suicidal behavior, such compering risk would most likely have diluted the association toward null. We have now acknowledged competing risk in the Discussion section (Page 17, Lines 378-381):

"*Fifth, given that parents of children with cancer might have a higher mortality than others, competing risk due to other causes of death might have diluted the observed positive association.*"

Competing risk methods used in the modeling cumulative incidence function, e.g., Fine-Gray subdistribution model, are useful for prediction in medical research. However, such methods have been shown by some studies to unlikely improve validity for causal inference and have little advantage for etiological studies like the present one [Wolbers M, Koller MT, Stel VS, Schaer B, Jager KJ, Leffondré K, Heinze G. Competing risks analyses: objectives and approaches. Eur Heart J. 2014 ;35(42):2936-41.] We have, therefore, decided not to perform

the suggested analysis at this time. We are however certainly willing to reconsider our position if the reviewer and editors would disagree with us.

Comment 3) The finding of slightly decreased risk of parental suicidal attempt more than seven years after a child's cancer diagnosis (HR, 0.86; 95%CI: 0.75-0.98) is difficult to comprehend. Apart from all the explanations in the discussion, the authors need to make sure the statistical analyses are robust, especially with the time-dependent and competing risk analyses.

Response:
Thank you for the comment. We hereby ensure that all statistical analyses are robust. Regarding the time-dependent and competing risk analyses, please kindly refer to our responses above.

Regardless, in light of the reviewer's comment and a suggestion from the editors, we have now toned down the statement about a "decreased" risk of suicide attempt from 7 years after cancer diagnosis of the child, but rather say that "the increased risk did not maintain beyond seven years after a child's cancer diagnosis", throughout the manuscript.

In Abstract (Page 3, Lines 32-35):
"*The increased risk did not, however, maintain beyond seven years after cancer diagnosis (HR, 0.86; 95%CI: [0.75, 0.98]; p=0.03) and there was no altered risk of parental death by suicide any time after the child's cancer diagnosis.*"

In Results (Page 12, Lines 261-264):
"*We observed an increased risk of suicide attempt among the exposed parents during the first seven years after a child cancer diagnosis, but the increased risk did not maintain thereafter in either the unadjusted **(Supplementary S2A Fig)** or the adjusted **(Fig 1A)** model.*"

In Results (Page 13, Lines 285-288):
"*The lack of increased risk of suicide attempt beyond seven years after a child cancer diagnosis was, on the other hand, mainly attributable to fathers, older parents, as well as parents without a history of cancer or a family history of psychiatric disorder.*"

In Discussion (Page 14, Lines 313-315):
"*There was no increased risk of parental suicide attempt later during follow-up, and there was no altered risk of parental death by suicide any time after a child cancer diagnosis.*"

In Discussion (Page 15, Lines 340-343):
"In contrast to an increased risk of parental suicide attempt during the first years after a child cancer diagnosis, we observed no increased risk of parental suicide attempt later during the follow-up and no altered risk of parental death by suicide any time after a child cancer diagnosis."

*** Reviewer #2:

This manuscript reports an investigation of the association between childhood cancer and suicide mortality/attempt in parents using data from two countries. My comments are as follows:

Comment: 1. The sibling comparison obviously has some limitations, in that there's presumably a reasonably high level of shared direct exposure (people going through the trauma of a niece or nephew with cancer) which isn't usually what's wanted in this technique. It could therefore represent 'over-adjustment' and result in overly conservative estimates. Although this doesn't explain the observed persistence of the findings of interest, I think this point is worth making in the Discussion.

Response:
Thank you for the great comment and we agree, completely. We have now discussed this in the Discussion section (Page 16, Lines 366-369):

"*However, as siblings of the exposed individuals also experienced, to different extent, the psychological distress of having a niece or nephew with a cancer diagnosis, the results of the sibling comparison may also represent an underestimation of the real association (i.e., overmatching).*"

Comment: 2. I think the Discussion ought to acknowledge the limitation of the cancer aggressiveness measure, since this was aggregated by type of cancer, and didn't take into account individual prognostic markers such as stage at presentation.

Response:
We agree and have now discussed this in the Discussion section (Pages 16-17, Lines 370-372):

"*Second, given a lack of information on prognostic markers of cancer (e.g., cancer stage at diagnosis), we classified cancer aggressiveness based on cancer type, which might have led to some misclassification.*"

Comment: 3. It would be helpful to have some information in the Discussion on the likely comprehensiveness of outcome capture from the diagnostic codes – presumably other research has investigated register data for this (i.e., coding accuracy for 'suicide attempt')

Response:
Thank you for the good comment. The procedure (including register resource and ICD codes) to identify suicide attempt has been widely used in previous studies. In Sweden, a validation study [Ludvigsson JF, Andersson E, Ekbom A, Feychting M, Kim JL, Reuterwall C, et al. External review and validation of the Swedish national inpatient register. BMC Public Health. 2011;11:450.] showed a positive predictive value (PPV) of 85%-95% for majority of the diagnoses in the Swedish Patient Register, including accidents and injuries. In Denmark, the PPV of identifying suicide attempt using ICD-10 codes was estimated to be around 82% [Randall JR, Roos LL, Lix LM, Katz LY, Bolton JM. Emergency department and inpatient coding for self-harm and suicide attempts: validation using clinician assessment data. Int J Methods Psychiatr Res. 2017 Sep; 26(3)]. The accuracy of death by suicide in Swedish Causes of Death Register and the Danish Causes of Death Register is also high, with a 81% and 90% agreement of the classification of suicide between the officially recorded death and

re-evaluation by experts in Sweden and Denmark, respectively[Ingvild Maria Tøllefsen, Karin Helweg-Larsen, Ingemar Thiblin, Erlend Hem, Marianne C Kastrup, Ullakarin Nyberg, Sidsel Rogde, Per-Henrik Zahl, Gunvor Østevold, and Øivind Ekeberg. Are suicide deaths under-reported? Nationwide re-evaluations of 1800 deaths in Scandinavia. BMJ Open. 2015;5(11):e009120].

We have now added this information in the Discussion section (Page 16, Lines 356-359):

"*The positive predictive value (PPV) of using register-based definitions of suicide attempt was shown to be high in the Swedish and Danish registers (above 80%)[18, 31]. The accuracy of register-based definitions of death by suicide is also high, with a reported 81% and 90% agreement after expert re-evaluation in Sweden and Denmark, respectively[32].*"

Comment: 4. It rather looks as if '7 years since diagnosis' as an outcome definition was applied following visual inspection of the incidence pattern rather than a priori, so I think this needs an appropriate note of caution, particularly when it's portrayed as the main finding in the abstract. This element of the analysis felt quite exploratory and it didn't help that it was allowed to vary between population and sibling comparisons.

Response:
Thank you for the great comment. As we calculated the HR at each year of follow-up, we found that HR became below 1 after seven years of follow-up. Regardless, we agree with the reviewer that the 7-year cut-off is potentially study specific, and have now toned down about this cut-off throughout the manuscript including the Abstract.

In Abstract (Page 3, Lines 38-40):
"*In this study, we observed an increased risk of parental suicide attempt during the first years after a child's cancer diagnosis, especially when the child was diagnosed during childhood, or with an aggressive or fatal form of cancer.*"

In Discussion (Page 15, Lines 340-343):
"In contrast to an increased risk of parental suicide attempt during the first years after a child cancer diagnosis, we observed no increased risk of parental suicide attempt later during the follow-up and no altered risk of parental death by suicide any time after a child cancer diagnosis."

Comment: 5. I feel that the lack of suicide mortality difference should be in the abstract, as this was one of the primary comparisons. However, some consideration would be helpful in the Discussion on the statistical power for this (i.e., what hazard ratio (for example, over the initial 7 years for consistency) would have been detectable from the number of events and sample size).

Response:
Thank you for the comment. We have now added results on death by suicide in the Abstract (Page 3, Lines 40-41):

"*There was however no altered risk of parental death by suicide at any time after a child's cancer diagnosis.*"

We also discussed statistical power in the analysis of death by suicide in the Discussion section (Page 17, Lines 376-378):

"*Forth, although our study had a binational study population and a large sample size, we had, regardless, relatively limited statistical power in the analysis of death by suicide given the rareness of this event.*"

Comment: 6. The presence or not of other offspring would seem to be an obvious factor influencing risk of attempted suicide in the parent. Was there no possibility of ascertaining sibling numbers for index children as a covariate?

Response:
Thank you for the great comment and we agree. Given the short time promised to perform the revision, we are currently unable to perform this analysis. However, we discussed about this possibility in the Discussion section (page 17, lines 381-384):

"*Sixth, as the presence of other children has been shown to alleviate grief of parents undergoing stressful life events, e.g., bereavement of a child due to death[33, 34], the association of child cancer diagnosis with parental suicidal behavior might vary for parents with or without other children. Future studies are therefore warranted to test such a hypothesis.* "

7. On page 6 - I don't think 'a cancer child' is appropriate terminology.

Response:
Thank you for spotting this. We have now replaced the expression by "child with cancer" or "children with cancer" throughout the manuscript.

*** Reviewer #3:

Comment: This manuscript details the use of two large, population-based cohorts to examine the risk of suicidal behavior among parents of children with a cancer diagnosis. This is an important topic of study given the psychological toll on any caregiver of individuals with medical conditions, and in particular parents. Although the study is of course limited to observational data, the authors employ two methods to try to facilitate causal inference: a matching approach and a comparison to the parents' siblings (the latter to address the possibility that some familial factors could jointly increase risk of cancer [passed down to the child] and suicidality [expressed by the parent]). The large sample sizes generally provide statistical power that is unavailable in other studies, and the use of national registry data ensures reasonably consistent, objective, clinically-relevant measures. Another strength of the study is that the authors are able to adjust for potentially important confounders, such as parental psychiatric history.

Response:
Thank you for the encouraging comments.

Comment: My primary concern is that the authors provide little justification for some of the decisions they made with regard to coding, which leads to questions about whether results are cherry-picked. For example, no explanation is given for splitting the child's cancer into three categories (CNS, hematological malignancy, and other), or for binning parental age into <40, 41-60, 60+. Were these categories pre-specified, or determined only after conducting preliminary analyses (which could be a problem, though could also be justified)?

Response:
Thank you for the comment. All analyses were pre-specified and not data-driven. We have now submitted the analysis protocol as a Supplementary Information, together with the revised manuscript.

In the Methods section (Page 6, Lines 113-114):

"*The prespecified analysis plan of the present study is presented in the **Supplementary S1 Text**.*"

In terms of cancer types as hematological malignancies and CNS cancers are the most common types of cancer in early life. For instance, they make up 2/3 of all cancers during childhood, we decided to classify cancer types by hematological malignancies, CNS cancers, and other types of cancer. We have now clarified this in the Methods section (Page 8, Lines 153-156):

"*As cancers in central nervous system (CNS) and hematological malignancies are the most common types of cancer among young individuals, accounting for around two thirds of all childhood cancers for instance, in addition to any cancer, we classified cancers into CNS cancer, hematological malignancy, and other type of cancer.*"

We classified parental age at <40, 41-60, and 60+ because the median age of the parents at cohort entry was 56 in the population comparison, and 51/49 in the sibling comparison. We have now also clarified this in the Methods section (page 10, lines 213-219):

"To assess the effect of parental characteristics, we performed stratified analyses by sex, age at the index date (<40, 40 to 60, or >60 classified according to median values of the study participants in the population and sibling comparisons), calendar year of the index date (<1990, 1990 to 1999, 2000 to 2009, or ≥2010), country of residence, household income, marital status, highest attained educational level, history of cancer, history of psychiatric disorder, and family history of psychiatric disorder."

Comment: Additional comments are noted below.

1) Please change "completed suicide" to "suicide" or "suicide death" throughout (see https://eur01.safelinks.protection.outlook.com/?url=https%3A%2F%2Fwww.camh.ca%2F-%2Fmedia%2Ffiles%2Fwords-matter-suicide-language-guide.pdf&data=05%7C01%7Cqianwei.liu%40ki.se%7C2a505f0d39e8411da46108dbc42281c2%7Cbff7eef1cf4b4f32be3da1dda043c05d%7C0%7C0%7C638319424869850059%7CUnknown%7CTWFpbGZsb3d8eyJWIjoiMC4wLjAwMDAiLCJQIjoiV2luMzIiLCJBTiI6Ik1haWwiLCJXVCI6Mn0%3D%7C3000%7C%7C%7C&sdata=3XUCjmAde5g9yvMHgwWGjjA06VuASppuGeC1KJV3idM%3D&reserved=0 and https://eur01.safelinks.protection.outlook.com/?url=https%3A%2F%2Fpsychology.org.au%2Fpublications%2Finpsych%2F2013%2Ffebruary%2Fbeaton&data=05%7C01%7Cqianwei.liu%40ki.se%7C2a505f0d39e8411da46108dbc42281c2%7Cbff7eef1cf4b4f32be3da1dda043c05d%7C0%7C0%7C638319424869850059%7CUnknown%7CTWFpbGZsb3d8eyJWIjoiMC4wLjAwMDAiLCJQIjoiV2luMzIiLCJBTiI6Ik1haWwiLCJXVCI6Mn0%3D%7C3000%7C%7C%7C&sdata=aLRZGS52M9rzh0YDFGclfWtM0pYiFRwudMTHoDcG3BQ%3D&reserved=0).

Response:
We have now replaced "completed suicide" with "death by suicide" throughout the manuscript, as suggested by the reviewer and the academic editor.

Comment: 2) When describing exclusion criteria, I recommend coming up with a different way to describe parents who had a child receive a cancer diagnosis prior to the onset of observation: "cancer child" is not ideal.

Response:
Thank you for the comment. We have now revised this in the Methods section (Pages 6-7, Lines 119-123):

"As we aimed to study newly diagnosed cancer in a child and the first-onset parental suicide attempt or death by suicide, parents that had a child diagnosed with cancer (N=283 in Denmark and N=149 in Sweden) or had a suicide attempt (N=998 in Denmark and N=1,169 in Sweden) before the study period were excluded from the main analysis."

We have also replaced the expression "cancer child" with "child with cancer" or "children with cancer" throughout the manuscript. Thank you for spotting this.

Comment:3) Further justification for including "deliberate self-harm" in identifying suicide attempt cases is necessary. That terminology typically refers to non-suicidal self-injury, which differs etiologically and epidemiologically from suicide attempt.

Response:

Thank you for the great comment. In the Danish registers, we included codes for "deliberate self-harm" to identify suicide attempt, because suicide attempt is sometimes recorded as “deliberate self-harm”. A previous study showed that ICD codes, combined with the contact reason codes of "deliberate self-harm", performed well in identification of suicide event. We have now clarified this in the Method section (Page 8, Lines 166-168):

“*To better identify suicide attempt, we additionally included the reason for contact code ‘4’ (deliberate self-harm) in the identification of suicide attempt in Denmark, as suggested[19].*”

Comment:4) Some covariates are time-varying (e.g., household income). Were these modeled as time-varying covariates, or was status at beginning of the observation period used? If the latter, were time-dependent coefficients used?

Response:
Thank you for the comment. All covariates were used as time-fixed variables at the date of cohort entry (i.e., date of cancer diagnosis of a child for the exposed parents and their matched unexposed parents or siblings). We did not consider time-varying covariate as changes in some of the covariates (e.g., household income) might be secondary to the exposure (cancer diagnosis of a child). We have now clarified this in the Methods section (page 10, lines 219-221):

“*As some of the covariates might change secondary to the exposure (i.e., cancer diagnosis of a child), all covariates were ascertained at the index date and used as time-fixed variables in the analyses.*”

Comment: 5) What was the rationale for how "cancer type" was split into three groups (CNS cancer, hematological malignancy, other)?

Response:
Thank you for the comment. As hematological malignancies and CNS cancers are the most common types of cancer in early life, e.g., hematological malignancies and CNS cancers account for around 2/3 of all cancers during childhood, we classified types of cancer as hemtaological maligiancy, CNS cancer, and other types of cancer. We have now clarified this in the Methods section (Page 8, Lines 153-156):

“*As cancers in central nervous system (CNS) and hematological malignancies are the most common types of cancer among young individuals, accounting for around two thirds of all childhood cancers for instance, in addition to any cancer, we classified cancers into CNS cancer, hematological malignancy, and other type of cancer.*”

Comment: 6) The authors state that they observed increased risk of parental suicide attempt in the first 7 years after the offspring's cancer registration, and decreased risk thereafter. Based on the figure, this seems a bit misleading, as confidence intervals appear to always include 1 (the null). This is inconsistent with results reported in Table 2, which indicate that CIs did not include 1 when the HRs were based on the first 7 years versus thereafter. Perhaps this is merely an issue with how the splines were placed for the figure, but more explanation is warranted for the sake of transparency.

Response:

Thank you for the comment. The reviewer is correct that, in the figure, the confidence intervals are often times including the null. We suspect that this might be due to limited statistical power to estimate individual time points during the follow-up. We therefore provided average HR for the entire follow-up as well as for two time-windows during the follow-up in Table 2. The reviewer is also correct that the placement of splines might also contribute.

Regardless, we have now added description on how the splines were placed in the Methods section (Pages 9-10, Lines 195-198) for the sake of transparency:

"*In all flexible parametric survival models, a spline with 5 degrees of freedom (4 intermediate knots and 2 knots at each boundary, placed according to quintile distribution of events) was used for the baseline rate, while a spline with 3 degrees of freedom was used for the time-varying effect.*"

Comment: 7) The authors might consider moving discussion of the results around suicide death to the Supplement; despite the large sample size, suicide death is sufficiently rare that even this study might be underpowered to detect effects.

Response:
Thank you for the comment. As death by suicide is an important outcome of this study, and the comparison of results between suicide attempt and death by suicide is one of the a priori determined aim of the study, we would prefer to keep the discussion of these results in the main text. Our decision is also motivated by the comments from the editors and other reviewers.

However, we do agree with the reviewer that the results of death by suicide should be interpreted with caution given the limited statistical power. We have added discussion on this in the Discussion section (page 17, lines 376-378):

"*Forth, although our study had a binational study population and a large sample size, we had, regardless, relatively limited statistical power in the analysis of death by suicide given the rareness of this event.*"

Comment: 8) Little note is made of the relatively small effect sizes observed and the degree to which HRs are attenuated in the sibling analysis. This seems to warrant additional discussion/explanation, particularly for readers who are less familiar with how studies including a family design can be used to account for some confounders.

Response:
Thank you for the good suggestion. We have now added discussion in this regard in the Discussion section (Page 16, Lines 365-366 and page 16, lines 366-369):

"*The diminished estimates observed in the sibling comparison could partly speak for residual confounding due to shared familial factors.*"

"However, as siblings of the exposed individuals also experienced, to different extent, the psychological distress of having a niece or nephew with a cancer diagnosis, the results of the

sibling comparison may also represent an underestimation of the real association (i.e., overmatching)."

Comment: 9) The Discussion could be improved if the authors provide some ideas of the implications of their findings. For example, should parents of children <18 who receive a cancer diagnosis be provided with referrals to counselors/therapists who can help them deal with the psychological stress that attends their situation? Given the audience of PLoS Medicine, I imagine that clinical recommendations would be broadly appreciated. Furthermore, this addition would speak to the journal's guideline that papers have "clear implications for patient care, public policy, or clinical research agendas."

Response:
Thank you for the comment. We have now discussed the implications of our findings both in the Abstract and in the Discussion section of the manuscript:

In Abstract (Page 3, Lines 41-43):
"Our findings suggest extended clinical awareness of suicide attempt among parents of children with cancer, especially during the first few years after cancer diagnosis."

In the main text (Page 17, Lines 393-396):
"*Our findings suggest the need of extended clinical awareness for the risk of suicide attempt among parents of children with cancer, primarily during the first years after cancer diagnosis. Vigilance and interventions for severe psychological distress and psychiatric symptoms might be important and effective.*"
