# Supplementary material for: Suicide attempt and death by suicide among parents of young individuals with cancer: A population-based study in Denmark and Sweden
Source: PLoS Med. 2024 Jan 16;21(1):e1004322. doi: 10.1371/journal.pmed.1004322 (PMC10791002; doi:10.1371/journal.pmed.1004322)
Supplement: Attachment — Submitted filename: Responses to reviewer comments.docx [file pmed.1004322.s010.docx]

**Requests from Editors:**

**GENERAL**

Thank you for your detailed and considered responses to previous editor and reviewer comments. Please see below for further revisions required prior to publication.

**Response:**

Thank you for the positive comment. We have now made further revisions to the manuscript, as suggested by you and the reviewers.

**Comment:** * We agree with the reviewer, please see below, that the claim of 7-years as being a critical end point is overstated in places, please temper these claims throughout (in particular the abstract and when reporting your results).

**Response:**

Thank you for the comment and we agree. We have now further tempered these claims throughout the manuscript, in particular the abstract and when reporting our results.

In Abstract (Page 3, Lines 27-33):

*“There was an increased risk of parental suicide attempt during the first years after a child’s cancer diagnosis (HR, 1.15; 95%CI, [1.03, 1.28]; p=0.01), particularly when the child was 18 or younger at diagnosis (HR, 1.25; 95%CI, [1.08, 1.46]; p=0.004), when the child was diagnosed with a highly aggressive cancer (HR, 1.60; 95%CI, [1.05, 2.43]; p=0.03), or when the child died due to cancer (HR, 1.63; 95%CI, [1.29, 2.06]; p<0.001). The increased risk did not, however, maintain thereafter (HR, 0.86; 95%CI: [0.75, 0.98]; p=0.03)...”*

In Results, (Page 12, Lines 260-266):

*“We observed an increased risk of suicide attempt among the exposed parents during the first years after a child cancer diagnosis, but the increased risk did not maintain throughout the follow-up in either the unadjusted (Supplementary S2A Fig) or the adjusted (Fig 1A) model. In the sibling comparison, we similarly observed an increased risk of suicide attempt during the first few years after a child cancer diagnosis but not throughout the follow-up, with (Fig 1B) or without (Supplementary S2B Fig) multivariable adjustment.”*

In Results (Page 13, Lines 278-286) (Please note that, as the average HR is time-period specific, we need to specify the seven years. However, we have tempered this by showing only once the number and are certainly willing to reconsider our position if the editors and reviewer would disagree with us.):

*“As the average HRs of suicide attempt were similar between the unadjusted and adjusted models, we present HRs with multivariable adjustment (Table 2). For instance, the mean HR of suicide attempt was 1.15 (95%CI, [1.03, 1.28]; p=0.01) during the first seven years after a child cancer diagnosis and 0.86 (95%CI, [0.75, 0.98]; p=0.03) thereafter. Such increased risk was mainly attributable to mothers, younger parents (60 or younger), earlier calendar years (before 2000), as well as parents without a history of cancer, a history of psychiatric disorder, or a family history of psychiatric disorder. The lack of increased risk of suicide attempt thereafter was, on the other hand, mainly attributable to fathers, older parents, as well as parents without a history of cancer or a family history of psychiatric disorder.”*

In Results (Page 13, Lines 288-294)

*“In the analysis of child cancer characteristics, we focused on suicide attempt and found that the increased risk of suicide attempt soon after a child’s cancer diagnosis was mainly observed in relation to a child cancer diagnosis at 18 or younger (HR, 1.25; 95%CI, [1.08, 1.46]; p=0.004), a child diagnosis of haematological malignancy (HR, 1.26; 95%CI, [1.02, 1.55]; p=0.03), a child diagnosis of highly aggressive cancer (HR, 1.60; 95%CI, [1.05, 2.43]; p=0.03), and when the child died from the cancer (HR, 1.63; 95%CI, [1.29, 2.06]; p<0.001) (Table 3).”*

**Comment:** ** Please provide further clarification as per the reviewer request (below) regarding parental age classifications.

**Response:**

Thank you the editor and the reviewer for being thorough. We originally classified parental age in these three groups, both according to the median age of the cohort as well as to represent younger, middle-age, and older parents. We have now clarified this in Methods (Page 10, Lines 212-214):

“*To assess the effect of parental characteristics, we performed stratified analyses by sex, age at the index date (<40, 40 to 60, or >60 classified to represent younger, middle-age, and older parents)...*”

**Comment:** REFERENCES

For in-text reference callouts please format as follows, for example, line 74, ‘…and financial challenges [2,3].’ Please note an additional space preceding the opening bracket and the absence of a space between the different citations. Please check and amend throughout.

**Response:**

Thank you for the comment. We have now formatted the references throughout the manuscript as suggested.

**Comment:** Line 302 – ‘…95% CI [1.002, 2.10];…’ any reason for reporting as such?

**Response:**

Thank you for the comment. Originally, we reported the lower limit of confidence interval as 1.002 to show that the lower limit did not include 1.00. However, we realized that we now present p values along with the confidence intervals throughout the manuscript and do not need to do such. We have now revised this into two decimals.

In Results (Page 14, Lines 299-300):

“*In the sibling comparison, the mean HR of suicide attempt was 1.45 (95%CI, [1.00, 2.10]; p=0.049)...*”

SOCIAL MEDIA

To help us extend the reach of your research, please detail any X (formerly Twitter) handles you wish to be included when we tweet this paper (including your own, your coauthors’, your institution, funder, or lab) in the manuscript submission form when you re-submit the manuscript.

**Response:**

We have now added the Twitter of our co-author Dang Wei (@Dangwei11).

Comments from Reviewers:

Reviewer #1: Thanks authors for their great effort to improve the manuscript. I am satisfied with the response and revision. No further issues needing attention.

**Response:**

Thank you for the encouraging comment.

Reviewer #2: My comments have been satisfactorily addressed.

**Response:**

Thank you for the encouraging comment.

Reviewer #3: The authors have made considerable revisions to the text, which overall result in a manuscript with improved clarity. However, some substantial concerns remain.

**Response:**

Thank you for the encouraging comment. We have now addressed your additional comments below and modified the manuscript further accordingly.

1) Despite feedback from the reviewers of the original submission, there remain references in the text to the seven-year mark as a critical end point to the elevated risk of suicide attempt. In one response to reviewers, the authors propose that statistical power is an issue that leads to confidence intervals overlapping 1. This could very well be the case, but if the HR estimate does not differ from 1, then it is not appropriate to suggest that there is an effect. Based on Figure 1, it appears that the CIs overlap 1 well short of seven years beyond the offspring's cancer registration. There is no reason to exaggerate the length of the observed associations, and references to the seven-year mark still need to be scaled back.

**Response:**

Thank you for the comment and we agree. We have now further tempered these claims throughout the manuscript, in particular the abstract and when reporting our results.

In Abstract (Page 3, Lines 27-33):

*“There was an increased risk of parental suicide attempt during the first years after a child’s cancer diagnosis (HR, 1.15; 95%CI, [1.03, 1.28]; p=0.01), particularly when the child was 18 or younger at diagnosis (HR, 1.25; 95%CI, [1.08, 1.46]; p=0.004), when the child was diagnosed with a highly aggressive cancer (HR, 1.60; 95%CI, [1.05, 2.43]; p=0.03), or when the child died due to cancer (HR, 1.63; 95%CI, [1.29, 2.06]; p<0.001). The increased risk did not, however, maintain thereafter (HR, 0.86; 95%CI: [0.75, 0.98]; p=0.03)...”*

In Results, (Page 12, Lines 260-266):

*“We observed an increased risk of suicide attempt among the exposed parents during the first years after a child cancer diagnosis, but the increased risk did not maintain throughout the follow-up in either the unadjusted (Supplementary S2A Fig) or the adjusted (Fig 1A) model. In the sibling comparison, we similarly observed an increased risk of suicide attempt during the first few years after a child cancer diagnosis but not throughout the follow-up, with (Fig 1B) or without (Supplementary S2B Fig) multivariable adjustment.”*

In Results (Page 13, Lines 278-286) (Please note that, as the average HR is time-period specific, we need to specify the seven years. However, we have tempered this by showing only once the number and are certainly willing to reconsider our position if the editors and reviewer would disagree with us.):

*“As the average HRs of suicide attempt were similar between the unadjusted and adjusted models, we present HRs with multivariable adjustment (Table 2). For instance, the mean HR of suicide attempt was 1.15 (95%CI, [1.03, 1.28]; p=0.01) during the first seven years after a child cancer diagnosis and 0.86 (95%CI, [0.75, 0.98]; p=0.03) thereafter. Such increased risk was mainly attributable to mothers, younger parents (60 or younger), earlier calendar years (before 2000), as well as parents without a history of cancer, a history of psychiatric disorder, or a family history of psychiatric disorder. The lack of increased risk of suicide attempt thereafter was, on the other hand, mainly attributable to fathers, older parents, as well as parents without a history of cancer or a family history of psychiatric disorder.”*

In Results (Page 13, Lines 288-294)

*“In the analysis of child cancer characteristics, we focused on suicide attempt and found that the increased risk of suicide attempt soon after a child’s cancer diagnosis was mainly observed in relation to a child cancer diagnosis at 18 or younger (HR, 1.25; 95%CI, [1.08, 1.46]; p=0.004), a child diagnosis of haematological malignancy (HR, 1.26; 95%CI, [1.02, 1.55]; p=0.03), a child diagnosis of highly aggressive cancer (HR, 1.60; 95%CI, [1.05, 2.43]; p=0.03), and when the child died from the cancer (HR, 1.63; 95%CI, [1.29, 2.06]; p<0.001) (Table 3).”*

2) The explanation for how parental ages were binned does not make sense to me. If the median age of the parents at cohort entry was 56, how does the middle bin of 41-60 line up with that?

**Response:**

Thank you the editor and the reviewer for being thorough. We originally classified parental age in these three groups, both according to the median age of the cohort as well as to represent younger, middle-age, and older parents. We have now clarified this in Methods (Page 10, Lines 212-214):

“*To assess the effect of parental characteristics, we performed stratified analyses by sex, age at the index date (<40, 40 to 60, or >60 classified to represent younger, middle-age, and older parents)...*”
